# Targeted Differentiation of Regional Ventral Neuroprogenitors and Related Neuronal Subtypes from Human Pluripotent Stem Cells

**DOI:** 10.1016/j.stemcr.2016.09.003

**Published:** 2016-10-06

**Authors:** Liankai Chi, Beibei Fan, Kunshan Zhang, Yanhua Du, Zhongliang Liu, Yujiang Fang, Zhenyu Chen, Xudong Ren, Xiangjie Xu, Cizhong Jiang, Siguang Li, Lin Ma, Liang Gao, Ling Liu, Xiaoqing Zhang

**Affiliations:** 1 Department of Neurosurgery, Shanghai Tenth People's Hospital, Neuroregeneration Key Laboratory of Shanghai Universities, Tongji University School of Medicine, 1239 Siping Road, Shanghai 200092, China; 2 Department of Regenerative Medicine, Tongji University School of Medicine, Shanghai 200092, China; 3 The School of Life Sciences and Technology, Tongji University, Shanghai 200092, China; 4 Tongji University Advanced Institute of Translational Medicine, 1239 Siping Road, Shanghai 200092, China; 5 The Collaborative Innovation Center for Brain Science, Tongji University, Shanghai 200092, China

**Keywords:** medial ganglionic eminence, floor plate, neural specification, human pluripotent stem cell, regenerative medicine

## Abstract

Embryoid body (EB) formation and adherent culture (AD) paradigms are equivalently thought to be applicable for neural specification of human pluripotent stem cells. Here, we report that sonic hedgehog-induced ventral neuroprogenitors under EB conditions are fated to medial ganglionic eminence (MGE), while the AD cells mostly adopt a floor-plate (FP) fate. The EB-MGE later on differentiates into GABA and cholinergic neurons, while the AD-FP favors dopaminergic neuron specification. Distinct developmental, metabolic, and adhesion traits in AD and EB cells may potentially account for their differential patterning potency. Gene targeting combined with small-molecule screening experiments identified that concomitant inhibition of Wnts, STAT3, and p38 pathways (3i) could largely convert FP to MGE under AD conditions. Thus, differentiation paradigms and signaling regulators can be integrated together to specify distinct neuronal subtypes for studying and treating related neurological diseases, such as epilepsy, Alzheimer's disease, and Parkinson's disease.

## Highlights

• EB and AD paradigms yield different ventral neuroprogenitors upon SHH patterning
• DA neurons of FP origin are generated in AD conditions upon SHH patterning
• Wnts/STAT3/p38 inhibition benefits MGE specification under AD conditions
• GABA and CHAT neurons of MGE origin are generated in EB or AD/3i conditions

## Graphical Abstract

**Figure:**
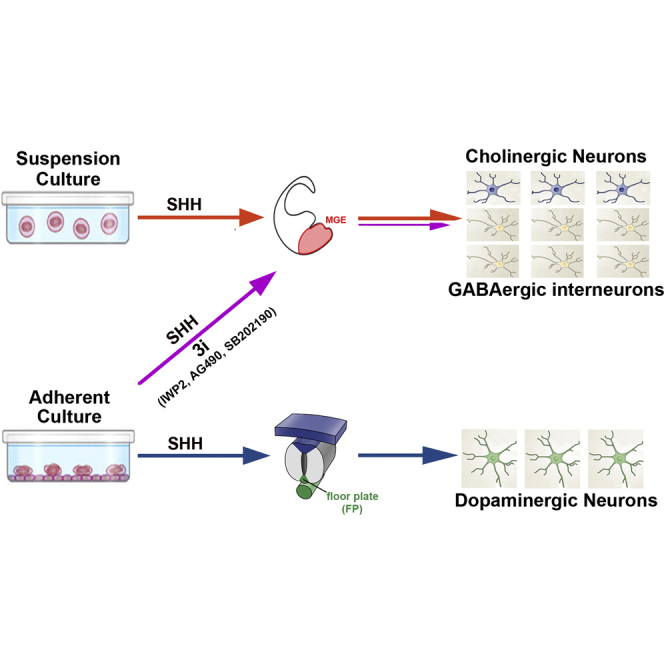

## Teaser

In this article, Zhang, Liu, Gao, and colleagues show that SHH-induced ventral neuroprogenitors under EB conditions are fated to MGE, while the AD cells are fated to FP. Wnts/STAT3/p38 pathways are closely involved in MGE and FP specification. GABA, cholinergic, and dopaminergic neurons of either MGE or FP origin could therefore be generated through integration of appropriate differentiation paradigms and signaling regulators.

## Introduction

To fully apply human pluripotent stem cells (hPSCs) to the study of neural development and disease, it is crucial to build efficient neural differentiation protocols with targeted regional identity. The embryoid body (EB) formation and dual-Smad inhibition-based adherent culture (AD) paradigms are the two most frequently applied neural differentiation systems for hPSCs (Chambers et al., 2009, Zhang et al., 2001). The EB formation method models the gastrulation period by suspension of detached hPSCs in the hPSC culture medium followed by the neural medium for neural lineage enrichment. The AD paradigm keeps hPSCs adherent, but triggers neural induction by applying inhibitors of both transforming growth factor β (TGF-β) and bone morphogenetic protein (BMP) signaling pathways.

With the EB protocol, hPSCs have been efficiently patterned to medial ganglionic eminence (MGE) and lateral ganglionic eminence (LGE) (Li et al., 2009, Liu et al., 2013, Ma et al., 2012). Functional basal forebrain cholinergic neurons and medium spiny γ-aminobutyric acid (GABA) neurons have been efficiently generated from these regional progenitors, which show remarkable therapeutic potentials in correcting behavioral abnormalities resembling those of Alzheimer's disease and Huntington disease (Liu et al., 2013, Ma et al., 2012, Yue et al., 2015). hPSCs has also been efficiently differentiated to floor plate (FP) and then midbrain dopamine (DA) neurons through the AD protocol, and the yielded DA neurons hold a promising role in correcting the phenotypes of Parkinson's disease (Fasano et al., 2010, Kriks et al., 2011, Steinbeck et al., 2015).

Although both EB and AD differentiation paradigms have been reported to be equally efficient in differentiating certain neuronal subtypes, such as spinal motor neurons and GABA interneurons (Chen et al., 2014, Du et al., 2015, Li et al., 2005, Li et al., 2008, Liu et al., 2013, Maroof et al., 2013, Qu et al., 2014, Wang et al., 2013, Xu et al., 2016), it remains unclear whether both EB and AD neural differentiation protocols are suitable for specifying all types of regional progenitors or whether they have any bias. Here, we systematically compare both protocols and offer a reproducible way to generate various ventral neuroprogenitors and related neuronal subtypes through selection of appropriate differentiation paradigms and signaling inhibitors.

## Results

### Both EB and AD Neural Differentiation Paradigms Are Highly Efficient and Favor Anterior Neural Fates in the Absence of Exogenous Morphogens

For the EB protocol, hPSC aggregates were suspended in human embryonic stem cell (hESC) medium (hESCM) for 4 days to initiate differentiation and then switched to the neural induction medium (NIM) for neural lineage specification (Figure S1A). For the AD protocol, hPSCs were adherently cultured on Matrigel (or mouse embryonic fibroblasts [MEFs]) at low density and hESCM/NIM (1:1 ratio) was supplied to the cells together with SB431542 and LDN193189, inhibitors to block TGF-β and BMP signaling, to trigger neural differentiation (Figure S1B). As shown in Figures S1C and S1D, both protocols were highly efficient for neural differentiation of H9 hESCs as demonstrated by synchronized formation of columnar neuroepithelia and neurospheres, as well as typical neurons, roughly within 4 weeks. qPCR analyses showed that neural lineage genes *PAX6* and *SOX1* were upregulated significantly during differentiation, while mesendodermal genes *T* (*Brachyury*), *CXCR4*, *GATA6*, *SOX17*, *MESP1*, and *EOMES* were not present, suggesting the effectiveness of both neural differentiation protocols (Figures S1E and S1F). Without exogenous morphogen treatment, neural progenitors specified from both EB and AD paradigms expressed anterior genes, such as *FOXG1*, *EMX2*, and *OTX2*, but were completely lacking in *PAX7*, *EN1*, or *HOXB4*, hallmark genes specific for diencephalon, midbrain, and hindbrain identities, respectively (Joven et al., 2013, Simeone, 2002) (Figures S1G and S1H). Therefore, hPSCs adopt an anterior telencephalic neural fate by default under both EB and AD conditions.

### Efficient Generation of Regional Neural Progenitors by Patterning Morphogens with Both EB and AD Paradigms

**Figure 1:**
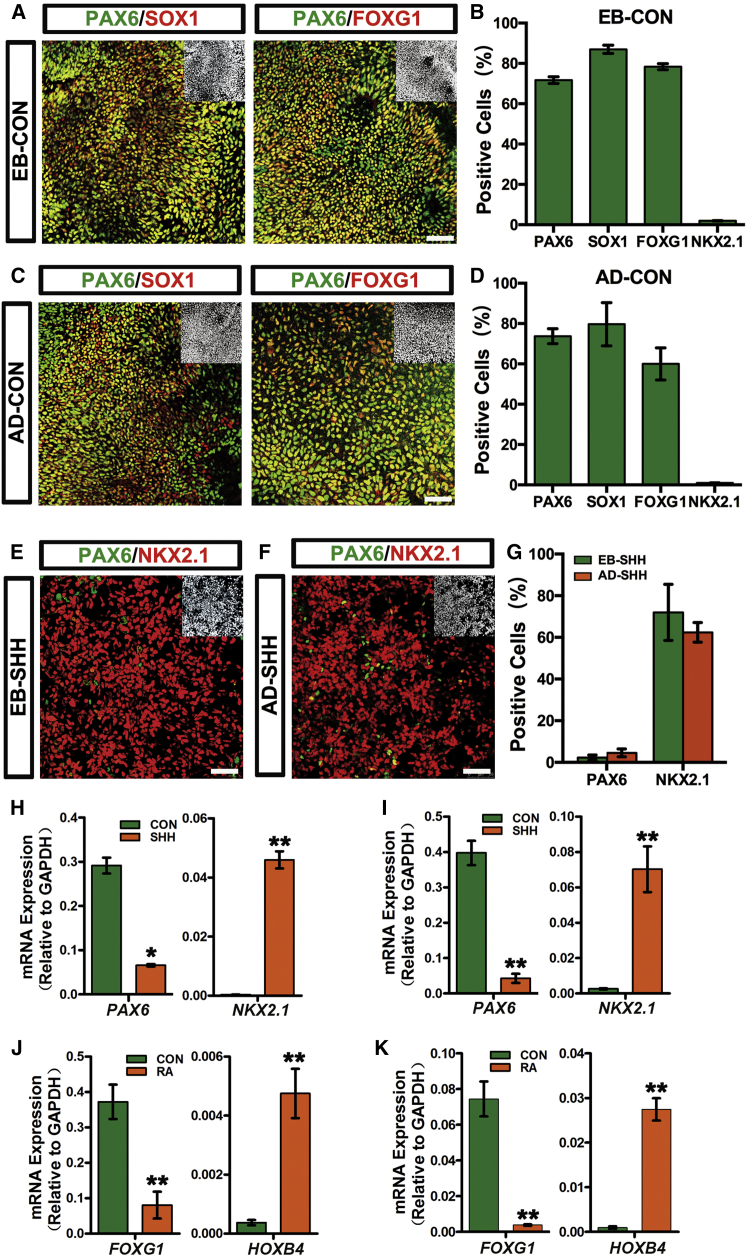
Neuroectoderm Generated with Both EB and AD Paradigms Holds Normal Patterning Potency along the A-P and D-V Axes (A–D) Without morphogen treatment, human neuroectoderm cells will take an anterior-dorsal fate as demonstrated by uniform PAX6, SOX1, and FOXG1 expression in committed regional neuroepithelia under both EB (day 17) (A) and AD (day 12) (C) conditions. Insets show Hoechst counterstaining of nuclei. Scale bars, 50 μm. Graphs (B) and (D) show the percentage of positive cells in (A) and (C), respectively. Data are presented as mean ± SEM of three independent experiments. (E–G) Confocal images show NKX2.1^+^/PAX6^−^ ventral progenitors yielded from both EB (E) and AD (F) differentiation paradigms. Insets show Hoechst counterstaining of nuclei. Scale bars, 50 μm. Graph (G) shows the percentage of positive cells in (E) and (F). Data are presented as mean ± SEM of three independent experiments. (H and I) SHH treatment at days 10–17 in EB (H) or days 4–12 in AD (I) differentiated cells results in efficient ventralization as shown by increased *NKX2.1*, while *PAX6* expression is decreased. Data are presented as mean ± SEM of three independent experiments. Unpaired two-tailed Student's t test: ^∗^p < 0.05, ^∗∗^p < 0.01. (J and K) RA treatment at days 10–17 in EB (J) or days 4–12 in AD (K) differentiated cells results in efficient caudalization as shown by increased *HOXB4*, while *FOXG1* expression is decreased. Data are presented as mean ± SEM of three independent experiments. Unpaired two-tailed Student's t test: ^∗∗^p < 0.01. See also Figure S1.

The columnar neuroepithelia differentiated from H9 hESCs with either EB or AD paradigms later on retained PAX6, SOX1, and FOXG1 expression (Figures 1A–1D). Moreover, these cells completely lacked the expression of NKX2.1, a specific transcription factor expressed in ventralmost populations along the neural axis (Sussel et al., 1999, Xu et al., 2004), suggesting a rostral-dorsal regional identity being acquired in the absence of exogenous patterning morphogens under both EB and AD conditions (Figures 1B and 1D). Immunostaining and qPCR analyses showed that addition of sonic hedgehog (SHH) from day 10 to day 17 under EB conditions or from day 4 to day 12 under AD conditions depleted *PAX6*, while significantly inducing *NKX2.1* expression (Figures 1E–1I). This suggests that a ventral fate is induced by applying SHH and that both differentiation protocols are competent for regional patterning. Upon retinoic acid (RA) treatment, *FOXG1* was repressed and *HOXB4* was greatly induced in both differentiation systems (Figures 1J and 1K), suggesting efficient caudalization. Therefore, hESCs could be patterned into regional progenitors along both dorsal-ventral (D-V) and anterior-posterior (A-P) axes with either EB or AD paradigms.

### SHH Induces Distinct MGE versus FP Fate under EB and AD Conditions

Notably, the absolute expression levels of *FOXG1* in EB (Figure 1J) and AD (Figure 1K) cells under the control conditions various significantly. Again, the expression levels of *HOXB4* also showed a big difference after RA caudalization in EB and AD cells. It is therefore reasonable to postulate that the EB and AD cells tend to adopt a telencephalic fate based on the uniform FOXG1 protein expression, increased mRNA expression levels of *FOXG1*, *OTX2*, and *EMX2*, and minimal mRNA expression of *PAX7*, *EN1*, or *HOXB4* (Figures S1G, S1H, and 1A–1D), while the EB and AD cells may hold different sensitivities in response to RA-mediated caudalization (Figures 1J and 1K).

**Figure 2:**
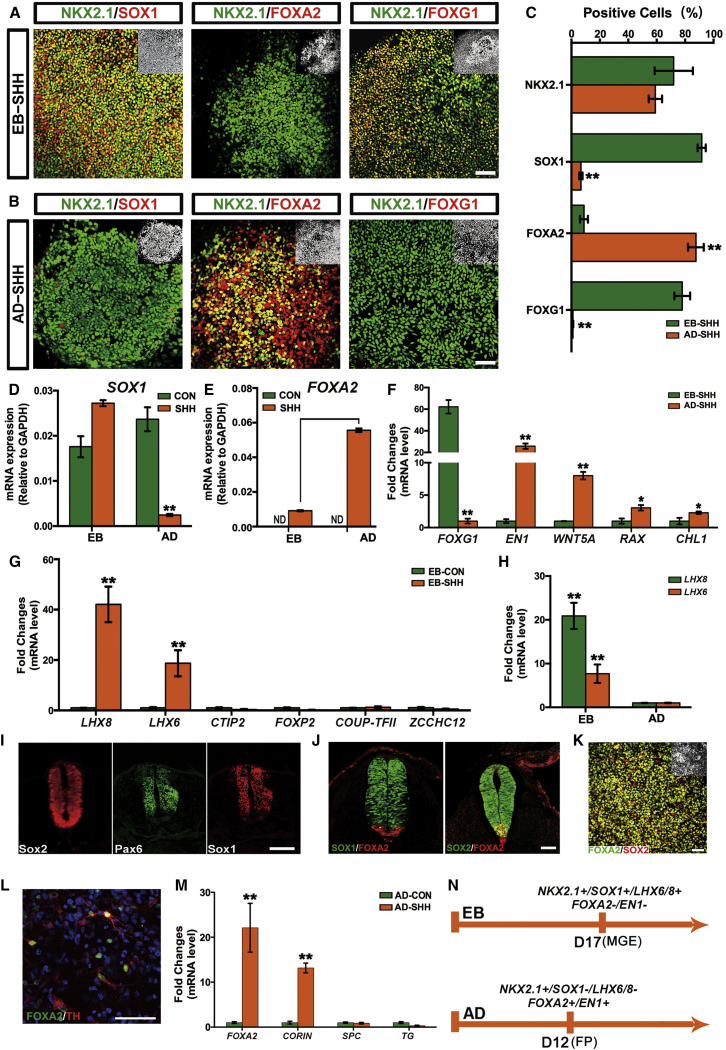
SHH Induces Distinct MGE versus FP Ventral Fates under EB and AD Conditions (A–C) Confocal images show expression of NKX2.1, SOX1, FOXA2 and FOXG1 at day 17 in EB (A) or day 12 in AD (B) differentiated neuroepithelia after SHH patterning. Insets show Hoechst counterstaining of nuclei. Scale bars, 50 μm. Graph (C) shows the percentage of positive cells in (A) and (B). Data are presented as mean ± SEM of three independent experiments. Unpaired two-tailed Student's t test: ^∗∗^p < 0.01. (D and E) mRNA expression of *SOX1* (D) and *FOXA2* (E) in both EB and AD differentiated neuroepithelia in the presence or absence of SHH exposure. Data are presented as mean ± SEM of three independent experiments. Unpaired two-tailed Student's t test: ^∗∗^p < 0.01. (F) In the presence of SHH exposure, cells differentiated under EB or AD conditions show differential mRNA expression levels of *FOXG1*, *EN1*, *WNT5A*, *RAX*, and *CHL1*. Data are presented as mean ± SEM of three independent experiments. Unpaired two-tailed Student's t test: ^∗^p < 0.05, ^∗∗^p < 0.01. (G) In EB cells with SHH treatment, neuroprogenitors show high expression of *LHX6* and *LHX8*, but no expression of *CTIP2*, *FOXP2*, *COUP-TFII*, and *ZCCHC12*. Data are presented as mean ± SEM of three independent experiments. Unpaired two-tailed Student's t test: ^∗∗^p < 0.01. (H) *LHX6* and *LHX8* are highly expressed in EB but not AD differentiated ventral neuroprogenitors. Data are presented as mean ± SEM of three independent experiments. Unpaired two-tailed Student's t test: ^∗∗^p < 0.01. (I) Confocal images of an E28 human embryo. Human FP cells are positive for SOX2, but negative for either PAX6 or SOX1. Scale bar, 50 μm. (J) Confocal images of an E9.5 mouse embryo. Mouse FP cells uniformly express FOXA2 and SOX2, but are negative for SOX1. Notochord cells are positive for FOXA2, but lack SOX1 and SOX2 expression. Scale bar, 50 μm. (K) The FP progenitors generated by SHH exposure under AD conditions are double-positive for FOXA2 and SOX2. Inset shows Hoechst counterstaining of nuclei. Scale bar, 50 μm. (L) The FP cells specified under AD conditions differentiate into TH^+^ neurons after long-term culture, some of which still retain FOXA2 expression. Scale bar, 50 μm. (M) *FOXA2* and *CORIN* mRNA is highly induced by SHH exposure under AD conditions, while *SPC* and *TG* can hardly be detected. Data are presented as mean ± SEM of three independent experiments. Unpaired two-tailed Student's t test: ^∗∗^p < 0.01. (N) Summary of distinct ventral progenitor fates generated under EB or AD conditions upon SHH treatment. See also Figure S2.

Regarding D-V patterning, the EB and AD cells also exhibited differential specification tendency. As shown in Figures 2A–2C, although NKX2.1 was equally expressed in SHH-patterned progenitors differentiated with both EB and AD methods, FOXA2 was selectively expressed in AD but not EB differentiated cells, suggesting that differential ventral fates were induced. We have previously demonstrated that under EB conditions, high levels of SHH treatment specifies an MGE fate (Li et al., 2009, Liu et al., 2013). With the EB paradigm, SHH-patterned cells expressed SOX1 and FOXG1, confirming a forebrain MGE identity (Figures 2A and 2C). In contrast, under AD conditions the FOXA2^+^ cells did not coexpress SOX1 or FOXG1 (Figures 2B and 2C), suggesting a non-MGE fate. qPCR data also confirmed the above expression pattern of SHH-treated EB and AD cells (Figures 2D–2F). Moreover, SHH-patterned AD cells expressed much higher levels of *EN1*, *WNT5A*, *RAX*, and *CHL1*, representative marker genes for midbrain and ventral diencephalon (Liu et al., 2000, Parr et al., 1993, Shimogori et al., 2010), at an expense of *FOXG1* expression as compared to SHH-treated EB cells (Figure 2F). *LHX6* and *LHX8*, key genes for GABA and acetylcholine neuronal progenitors developed from MGE (Flames et al., 2007, Manabe et al., 2005, Zhao et al., 2003), were highly induced in SHH-ventralized EB cells (Figure 2G). On the other hand, *CTIP2*, *FOXP2*, *COUP-TFII*, and *ZCCHC12*, hallmark genes related to LGE or caudal ganglionic eminence (Arber et al., 2015, Cambray et al., 2012), could hardly be identified in SHH-treated EB cells as revealed by qPCR analysis (Figure 2G). In contrast, under AD conditions *LHX6* and *LHX8* were not expressed in the ventralized AD progenitors (Figure 2H). All these data support our conclusion that SHH induced an MGE fate under EB conditions, while a non-MGE fate will be adopted in SHH-treated AD cells.

During primitive streak formation, FOXA2 is turned on and specifically expressed in the notochord, FP, and endoderm tissues along the longitudinal body axis (Ang et al., 1993, Placzek, 1995, Sasaki and Hogan, 1993). The most anterior tip of FOXA2-expressing cells is close to the ventral diencephalon, which also expresses NKX2.1 (Fasano et al., 2010, Placzek and Briscoe, 2005), while the FP at the midbrain and hindbrain regions is FOXA2^+^/NKX2.1^−^. In an embryonic day 28 (E28) human embryo, the notochord cells were SOX1^−^/PAX6^−^/SOX2^−^ and the FP cells SOX1^−^/PAX6^−^/SOX2^+^ (Figure 2I). In an E9.5 mouse embryo, the entire neural tube, including the roof plate, neuroepithelium, and FP, was SOX2^+^, while the FOXA2-expressing notochord cells were negative for SOX2 (Figure 2J). The FP cells uniformly expressed FOXA2 but lacked SOX1 expression (Figure 2J). Combined with these in vivo expression data, the AD ventral cells differentiated from hESCs were FOXA2^+^/SOX2^+^/SOX1^−^, proffering an FP fate (Figure 2K). Moreover, the AD ventral cells differentiated into FOXA2^+^/TH^+^ dopamine neurons in a long-term culture, further supporting their neural and FP identity (Figure 2L). *CORIN*, another representative FP characteristic gene, was also highly expressed in the AD ventral cells. Moreover, *surfactant protein C* (*SPC*) and *thyroglobulin* (*TG*), specific genes for lung and thyroid (Longmire et al., 2012, Xi et al., 2012), respectively, were not induced in the SHH-treated AD cells (Figure 2M).

We next repeated the above results in H1 hESC cell line and a human induced PSC (hiPSC) line (Hu et al., 2010). Again, SHH-treated EB cells expressed high levels of *FOXG1*, *SOX1*, *LHX6*, and *LHX8*, while SHH-treated AD cells expressed high levels of *FOXA2*, but almost complete lack of *FOXG1*, *SOX1*, *LHX6*, or *LHX8* expression (Figures S2A–S2H). Taken together, these observations suggest that after SHH treatment, hESCs and hiPSCs yield MGE progenitors under the EB differentiation conditions, while an FP fate is specified under AD conditions (Figure 2N). Since these FP progenitors expressed *EN1*, *WNT5A*, *RAX*, and *CHL1*, and some of the FOXA2^+^ cells also double-positive for NKX2.1 (Figures 2B and 2F), we therefore hypothesize that these FP progenitors derived under AD conditions bear a diencephalic or a midbrain regional identity.

### TGF-β/BMP Inhibition or Timing of SHH Exposure Could Not Account for the Variation of EB and AD Differentiation Paradigms

The aforementioned differences between AD and EB systems may result from either the application of TGF-β/BMP inhibitors or the timing of SHH treatment. To test these possibilities, we removed either LDN193189 or SB431542 from the AD differentiation system, and results showed that the majority of the cells remained FOXA2^+^/NKX2.1^+^/SOX1^−^ upon SHH patterning, indicating an FP identity (Figures S3A and S3B). To test whether earlier SHH exposure prefers to generate FP cells, SHH was added to the EB cells 1, 4, or 10 days post differentiation. However, *FOXA2* expression remained at low levels in all of these three time sets, although *NKX2.1* was equally induced (Figure S3C). Under AD conditions, SHH treatment at days 1, 4, or 7 equally induced an FP fate, illustrated by concomitant *FOXA2* and *NKX2.1* induction (Figure S3D). The AD cells were committed to a dorsal fate and lost their patterning potency after day 10, since no *FOXA2* or *NKX2.1* could be induced at the expense of *PAX6* expression thereafter (Figures S3D and S3E). Together, these results indicate that the differential potency in MGE versus FP patterning of EB and AD cells does not result from TGF-β/BMP inhibition or the timing of SHH exposure.

Both EB (Cunningham et al., 2014, Kim et al., 2014, Liu et al., 2013) and AD (Maroof et al., 2013) cultures have been applied for MGE specification from hPSCs. In the study by Maroof et al. (2013), the AD cells were treated with DKK1 to inhibit Wnts signaling and the cells patterned with SHH starting from day 10. This seems contradictory to what we propose above. To address this issue, we took our *β-catenin* knockout (KO) hESC lines (Chi et al., 2016, Liu et al., 2016b) and studied their D-V patterning under the AD culture conditions. Different from the wild-type control, *β-catenin* KO AD cells at day 10 were efficiently ventralized by SHH treatment, suggesting that blocking Wnts signaling extended the efficient patterning window for SHH (Figure S3F). *LHX6* and *LHX8* were also mildly increased under the AD culture conditions by SHH treatment in *β-catenin* KO hESCs (Figure S3G). Moreover, addition of SHH at a later time point seems to favor MGE specification because when SHH was applied to *β-catenin* KO AD cells at days 4, 7, or 10, the latter yielded a better outcome of MGE progenitors (Figures S3H–S3M). These data indicate that blocking Wnts/β-catenin signaling moderately improves MGE specification, but is not sufficient to completely convert FP to MGE under AD conditions.

### Transcriptional Control of FP and MGE Specification

**Figure 3:**
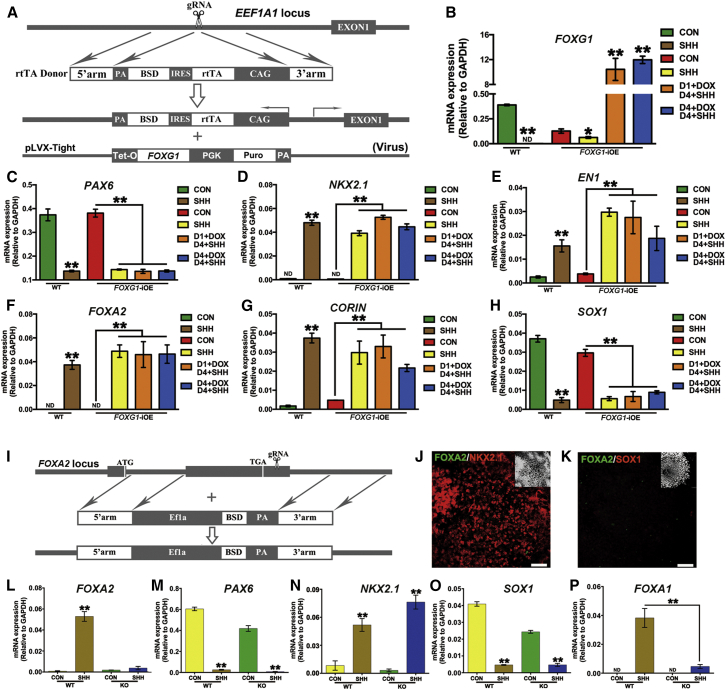
Targeting *FOXG1* and *FOXA2* Fails to Bring the AD Cells an MGE Identity (A) Schematic representation of the strategy in construction of *FOXG1*-iOE hESCs. (B–H) mRNA levels of *FOXG1*, *PAX6*, *NKX2.1*, *EN1*, *FOXA2*, *CORIN*, and *SOX1* in day-12 AD differentiated wild-type (WT) or *FOXG1*-iOE hESCs in the absence or presence of SHH. In some groups, doxycycline (Dox) is added to the *FOXG1*-iOE hESCs to induce exogenous *FOXG1* expression. Data are presented as mean ± SEM of three independent experiments. Unpaired two-tailed Student's t test: ^∗^p < 0.05, ^∗∗^p < 0.01. (I) Schematic representation of the strategy in construction of *FOXA2* KO hESCs. (J and K) Confocal images show NKX2.1^+^/SOX1^−^ FP-like cells induced in SHH-treated *FOXA2* KO cells under AD conditions. Insets show Hoechst counterstaining of nuclei. Scale bars, 50 μm. (L–P) *FOXA2*, *PAX6*, *NKX2.1*, *SOX1*, and *FOXA1* mRNA expression in day-12 AD differentiated wild-type or *FOXA2* KO hESCs in the absence or presence of SHH. Data are presented as mean ± SEM of three independent experiments. Unpaired two-tailed Student's t test: ^∗∗^p < 0.01. See also Figure S3.

FP and MGE show differential expression of FOXA2 and FOXG1, which play essential roles in FP and forebrain development, respectively. We next asked whether genetic manipulation of these transcription factors could switch the FP fate to an MGE identity under AD conditions. We constructed a *FOXG1*-inducible overexpression line (*FOXG1*-iOE) in hESCs (Figures 3A and 3B). *FOXG1*-iOE showed normal D-V patterning in the presence of SHH as demonstrated by decreased *PAX6* while *NKX2.1* expression increased (Figures 3C and 3D). However, overexpression of *FOXG1* could not convert the FP to an MGE fate, since the cells still showed equally high levels of *EN1*, *FOXA2*, and *CORIN* expression with relatively low *SOX1* expression (Figures 3E–3H). We then knocked out *FOXA2* in hESCs (Figure 3I). *FOXA2* KO cells were similarly patterned ventrally by SHH (Figures 3J and 3K). Moreover, the ventral cells still retained an FP-like but non-MGE identity, given their PAX6^−^/NKX2.1^+^/SOX1^−^ expression pattern (Figures 3J–3O). In the *FOXA2* KO cells, *FOXA1* was decreased but remained at a detectable level (Figure 3P). It remains to be investigated whether *FOXA1* and *FOXA2* play redundant roles in mediating FP development.

### Developmental, Metabolic, and Adhesion Properties Predetermine the FP versus MGE Specification

**Figure 4:**
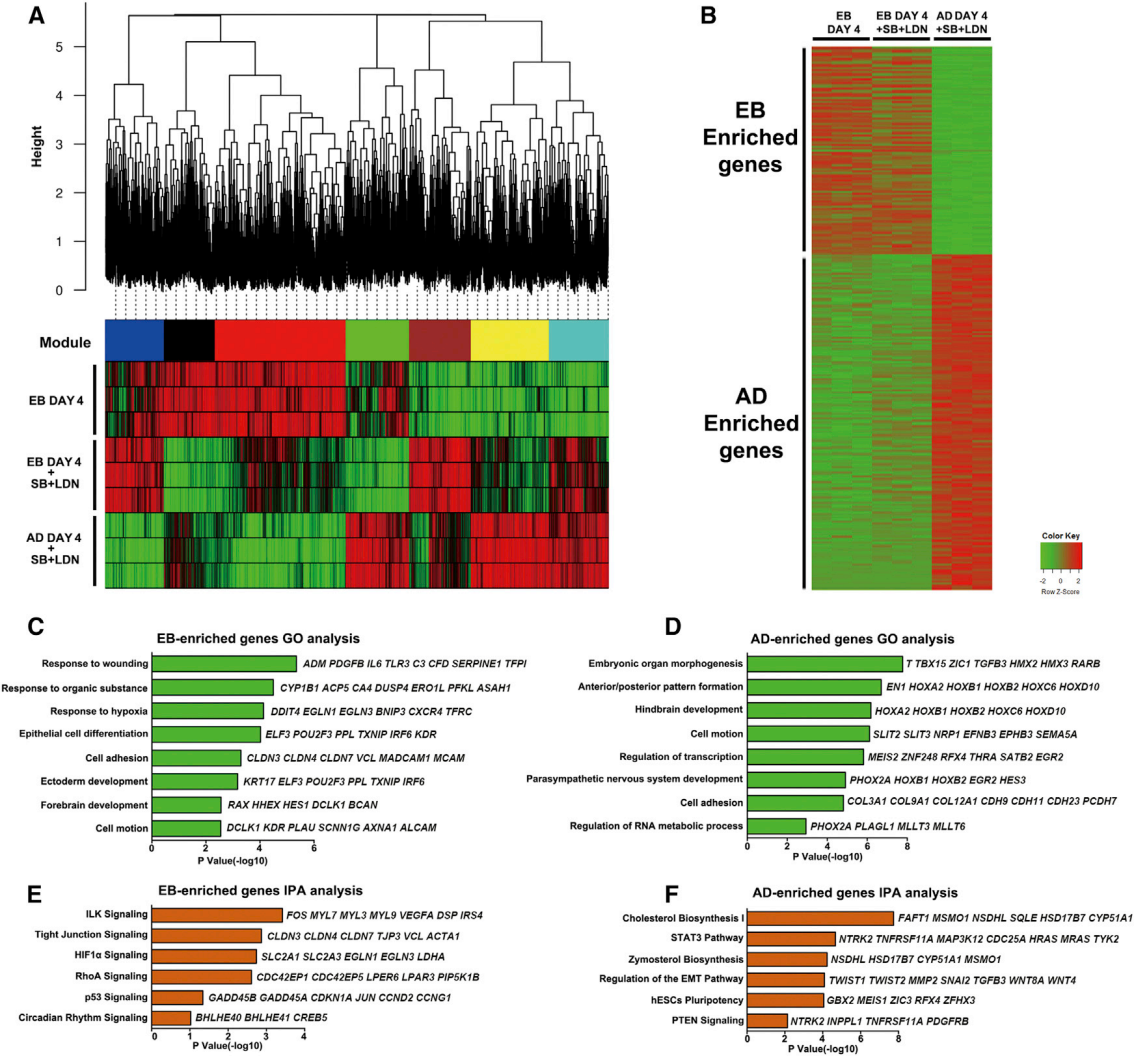
Transcriptome Profiling of EB and AD Cells Destined to Ventralize into MGE or FP (A) WGCNA analyses show isolated interconnected gene modules and module-trait correlation matrix demonstrated gene clusters enriched in either EB or AD cells. (B) Heatmap graph shows EB- and AD-enriched genes. (C–F) Gene ontology (C, D) and ingenuity pathway (E, F) analyses of EB- and AD-enriched genes.

To delineate the underlying mechanisms guiding FP and MGE specification, we profiled transcriptomes of early AD and EB cells destined to undergo distinct ventral fates before SHH treatment. We first adjusted the differentiation paradigm and tried to minimize the systematic differences between AD and EB protocols (Figures S4A and S4B). AD cells treated with SB431542 and LDN193189 for 4 days differentiated into FP, while EB cells treated with or without SB431542 and LDN193189 for 4 days mostly fated to MGE (Figures S4C and S4D). We therefore chose day-4 EB cells, day-4 EB cells with SB431542 and LDN193189, and day-4 AD cells with SB431542 and LDN193189 for mRNA-sequencing analyses (GEO: GSE82052). Through weighted gene coexpression network analyses (WGCNA) (Langfelder and Horvath, 2008), we isolated seven interconnected gene modules (Figure 4A). Module-trait correlation matrix demonstrated that these gene clusters showed differential gene expression enriched in either EB cells (blue and red modules) or AD cells (yellow and green modules) (Figure 4A). We found a total of roughly 600 genes which were specifically enriched in both day-4 EB cells and day-4 EB cells treated with SB431542 and LDN193189 (Figure 4B). Meanwhile, there were around 1,000 genes which showed significantly higher expression in AD cells (Figure 4B). Gene ontology biological process term enrichment of differentially expressed genes revealed that developmental traits, adhesion properties, and metabolic states showed prominent differences in EB and AD cells. EB cells showed upregulated epithelial genes including *KRT17*, *ELF3*, and *POU2F3* (Figure 4C). In contrast, AD cells expressed high levels of *T*, *TBX15*, and *ZIC1*, genes expressed at late-stage epiblast or primitive streak (Figure 4D). Moreover, mesenchymal genes, such as *SNAI2*, *TWIST1*, and *TWIST2*, were significantly expressed in AD cells (Figure 4D). EB cells expressed high levels of tight junction-related genes, while AD cells were enriched with genes encoding cadherin family proteins and collagens (Figures 4C and 4D). Meanwhile, EB cell-enriched genes were clustered in HIF1α and wound-healing pathways together with organic substance metabolism. On the other hand, AD cells showed more robust cholesterol and zymosterol biosynthesis activities and were also active in the STAT3 and phosphatidylinositol 3-kinase (PI3K)/PTEN signaling pathways correlated with nutrient sensing (Figures 4C–4F). Therefore, complex intracellular cell contexts and extracellular components work together and define the distinct differentiation potencies of the AD and EB cells upon SHH patterning.

### Generation of a Floor-Plate Reporter Line by Targeting of FOXA2

**Figure 5:**
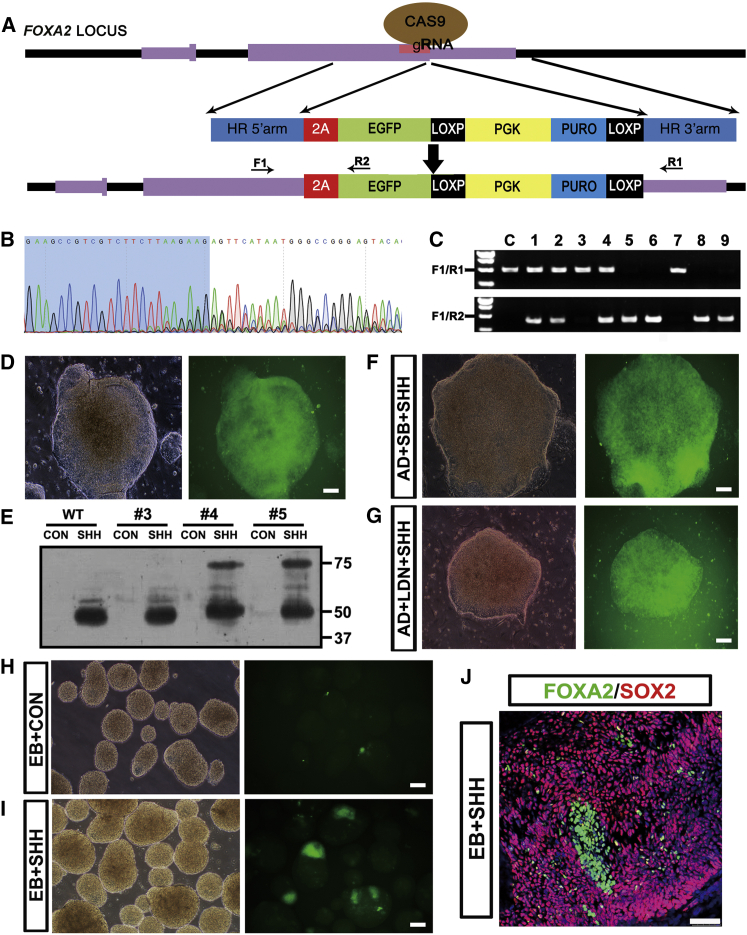
Generation of an FP Reporter Line by Targeting *FOXA2* (A) Donor DNA used for homologous recombination. Locations of primer sets for genotyping are marked with arrows. (B) Targeting efficiency of the designed gRNA is verified in HEK 293FT cells after transient transfection of the gRNA and Cas9 expression vectors. Sequencing of the PCR-amplified genomic DNA surrounding the targeting site identifies overlapped peaks, which represents non-homologous end-joining repair after correct DNA targeting. (C) Genotyping experiments show that seven out of nine randomly picked hESC colonies are successfully targeted. C, wild-type control; F1/R1, primer pair amplifies wild-type allele; F1/R2, primer pair amplifies homologous recombined allele. (D) The heterozygous *FOXA2*-2A-eGFP line (colony #1) shows uniform GFP expression 12 days after SHH exposure with the AD differentiation paradigm, recapitulating endogenous *FOXA2* expression. Scale bar, 50 μm. (E) Colonies #3, #4, and #5 are differentiated for 12 days with the AD differentiation paradigm in the presence or absence of SHH. FOXA2 (48 kDa) is only expressed in the SHH-treated groups. FOXA2-2A-eGFP fusion protein (75 kDa) is expressed in colonies #4 and #5, and a large population of FOXA2-2A-eGFP is cleaved at the 2A site as shown by slightly slower shifting bands compared with the endogenous FOXA2. (F and G) Removal of LDN193189 or SB431542 in the AD differentiation protocol does not affect FP specification as shown by similar GFP expression in a *FOXA2*-2A-eGFP reporter line induced by SHH. Scale bars, 50 μm. (H) *FOXA2*-2A-eGFP reporter line is differentiated under EB conditions for 20 days without extra patterning morphogens. No GFP expression is observed under the fluorescence microscope. Scale bars, 50 μm. (I) *FOXA2*-2A-eGFP reporter line is differentiated under EB conditions for 20 days and SHH is applied from day 10 to day 20. Only a small population of GFP-positive cells is seen under the fluorescence microscope. Scale bars, 50 μm. (J) Confocal image shows that the FOXA2-positive cells derived from the EB differentiated cells are SOX2 negative, indicating a notochord fate rather than FP. Scale bar, 50 μm. See also Figure S4.

To better monitor the different ventral progenitor fates, we constructed a *FOXA2*-2A-EGFP reporter line through homologous recombination (Figure 5A). The CRISPR/Cas9 system was used to make a double-strand break surrounding the stop codon region of *FOXA2*. Targeting efficiency of the guide RNA (gRNA) was tested in human embryonic kidney (HEK) 293FT cells 3 days after transient transfection of the gRNA and Cas9 vectors (Figure 5B). A homologous recombination donor plasmid was designed to incorporate the P2A-EGFP cassette just upstream of the stop codon and with the puromycin selection gene downstream. *FOXA2*-2A-EGFP targeting donor vector, gRNA, and Cas9 expressing vectors were coelectroporated into hESCs and puromycin-resistant colonies were selected. Retrieved colonies were then subjected to genomic DNA PCR analyses by using primer sets specifically targeting the wild-type allele and the recombined allele. Among the nine colonies picked up, colonies 1, 2, and 4 represented heterozygotes (*FOXA2*^EGFP/w^), and colonies 5, 6, 8, and 9 represented homozygotes (*FOXA2*^EGFP/EGFP^) (Figure 5C). We then chose colony 1 (*FOXA2*^EGFP/w^) for AD differentiation, and SHH induction initiated GFP expression from day 5. GFP was uniformly expressed in most of the cells 12 days post differentiation (Figure 5D). Western blotting analyses further demonstrated that most of the FOXA2-2A-EGFP fusion proteins were cleaved into FOXA2 and EGFP, coinciding with the cytoplasmic expression of GFP (Figure 5E). Neither removal of LDN193189 nor removal of SB431542 abrogated GFP expression induced by SHH under AD conditions (Figures 5F and 5G), consistent with previous results (Figure S3). In the SHH-treated EB group, a small population of cells were positive for GFP (Figures 5H and 5I), but these FOXA2^+^ cells were negative for SOX2 (Figure 5J). The FOXA2^+^/SOX2^−^ cells most likely had a notochord fate, further supporting the point that FP cells could hardly be generated under EB conditions. Therefore, the GFP expression pattern faithfully recapitulates FOXA2 expression, and the *FOXA2*^EGFP/w^ line is a reliable reporter system for human FP under AD conditions.

### The STAT3 and p38 MAPK Signaling Pathways Are Crucial for Floor-Plate Specification

To investigate the underlying mechanisms for proper MGE and FP specification, we carried out a small-molecule screening by targeting different signaling pathways in *FOXA2*^EGFP/w^ hESCs. The AD differentiation paradigm was accommodated to the 48-well culture plates. In total, 303 stem cell regulators and 80 kinase inhibitors purchased from the National Compound Resource Center (http://www.chemicallibrary.org.cn/11115.html) were added to the AD *FOXA2*^EGFP/w^ cells together with SHH at day 1. Bright-field and GFP images were taken at day 7 (Figure S5A). Among all the small molecules, nearly 110 drugs induced cell-growth arrest or cell death over the first several days. Within the remaining 270 drugs, we found seven small molecules that significantly repressed GFP intensity during AD differentiation. These drugs were cyclopamine (an SHH inhibitor), SB202190 (a p38 mitogen-activated protein kinase [MAPK] inhibitor), AG490 (a JAK2-STAT3 inhibitor), DAPT (a Notch inhibitor), ZM336372 (a Raf-1 inhibitor), KN62 (a CaMKII inhibitor), and LY294002 (a PI3K inhibitor). The observation that cyclopamine efficiently repressed EGFP expression ensured the feasibility of the current reporter system for signaling screening (Figures S5B and 5C).

**Figure 6:**
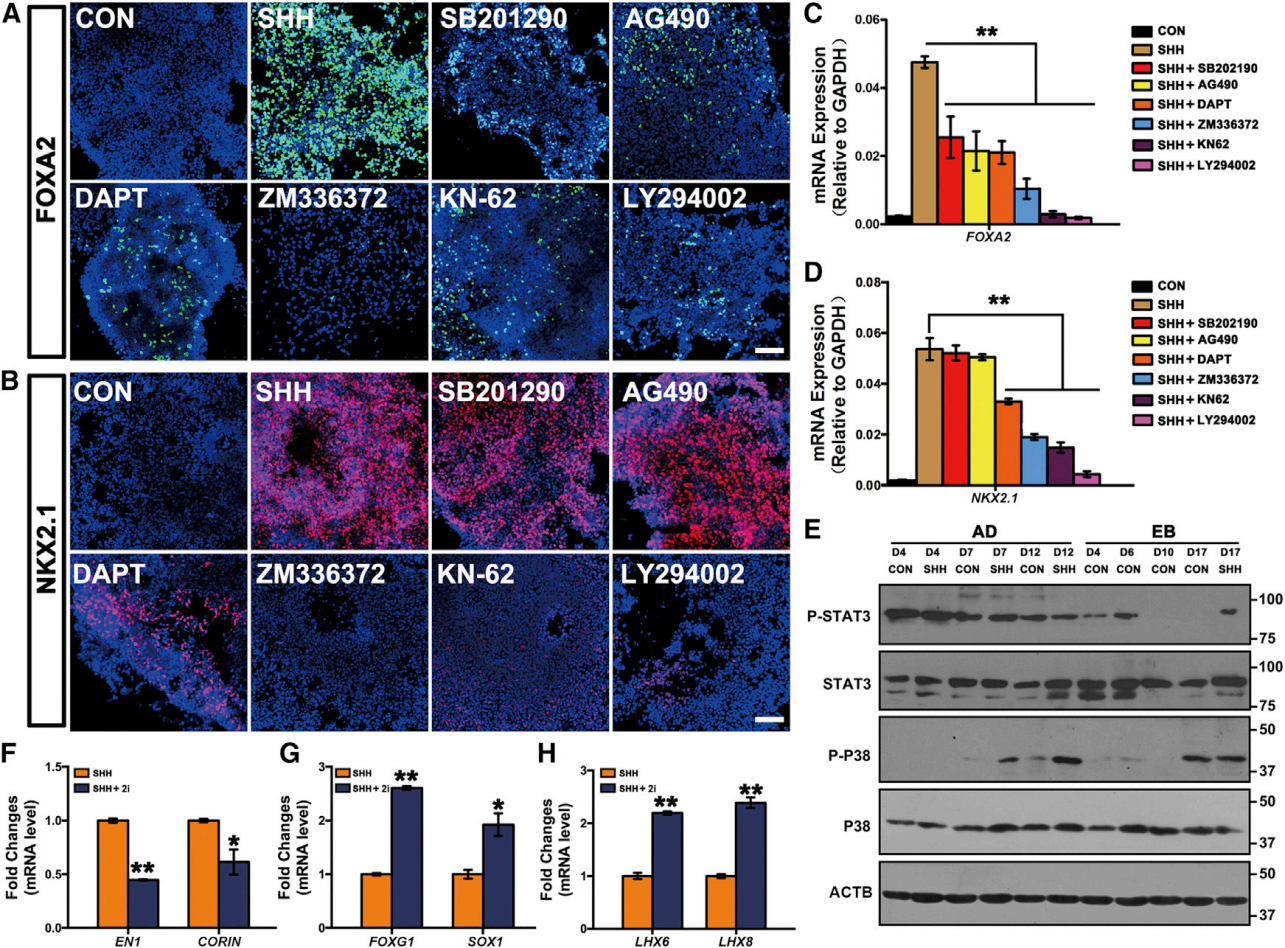
The STAT3 and p38 MAPK Signaling Pathways Are Crucial for Floor-Plate Specification (A and B) Confocal images show expression of FOXA2 (A) and NKX2.1 (B) in day-12 neuroepithelia differentiated under AD conditions in the presence of SHH and represented signaling inhibitors. Scale bars, 50 μm. (C and D) *FOXA2* (C) and *NKX2.1* (D) mRNA expression in day-12 neuroepithelia differentiated under AD conditions in control, SHH, or SHH plus represented signaling inhibitor groups. Data are presented as mean ± SEM of three independent experiments. Unpaired two-tailed Student's t test: ^∗∗^p < 0.01. (E) Western blot shows temporal activation of STAT3 and p38 MAPK along hESC differentiation under AD or EB conditions. (F–H) qPCR results show that combined AG490 and SB202190 treatment decreases *EN1* and *CORIN* (F), but increases *FOXG1* and *SOX1* (G) and *LHX6* and *LHX8* (H) expression after SHH exposure. Data are presented as mean ± SEM of three independent experiments. Unpaired two-tailed Student's t test: ^∗^p < 0.05, ^∗∗^p < 0.01. See also Figure S5.

Immunocytochemistry analysis and qPCR results confirmed that *FOXA2* protein and mRNA levels were repressed in the presence of all of the above inhibitors (Figures 6A and 6C). Among these small molecules, KN62 and LY294002 showed the highest robustness in repression of FOXA2 (Figures 6A and 6C). Meanwhile, *NKX2.1* and *SOX2* were also concomitantly repressed by these two inhibitors, suggesting that a non-neural fate was induced (Figures 6B, 6D, and S5D). Indeed, *VEGFR2*, a mesodermal marker, was highly expressed in KN62- or LY294002-treated cells (Figure S5E). These data indicate that the CaMKII and PI3K signaling pathways are required for neural induction. ZM336372 inhibited both *FOXA2* and *NKX2.1* expression, while *PAX6* was highly expressed even under SHH treatment (Figures 6A–6D and S5F). This suggests that Raf-1 signaling is required for SHH-triggered ventralization. DAPT also repressed both *FOXA2* and *NKX2.1* (Figures 6A–6D), indicating the involvement of the Notch pathway in proper FP development. Interestingly, SB202190 and AG490 inhibited *FOXA2* but maintained *NKX2.1* expression (Figures 6A–6D). This indicates that the activity of p38 MAPK and JAK2-STAT3 signaling pathways are crucial for FP specification, and blocking these two pathways could benefit MGE specification under AD conditions.

To confirm that FP and MGE fates could indeed be switched over by modulating the activity of p38 MAPK and JAK2-STAT3, we analyzed whether these pathways were activated during FP specification by western blotting. As shown in Figure 6E, STAT3 was highly phosphorylated at the early stage of AD, but not EB, differentiated cells. The protein expression of total STAT3 and the phosphorylated form of STAT3 dramatically decreased upon RA treatment in hESCs and hiPSCs (Figure S5G). Under AD conditions, SHH treatment greatly triggered p38 phosphorylation at days 7 and 12 post differentiation. These data strongly suggest that p38 MAPK and JAK2-STAT3 signaling pathways may play important roles in FP specification. The differential activation pattern of these two pathways also suggests that their roles in FP specification may vary. We next treated the AD cells with both AG490 and SB202190 (hereafter referred to as 2i) from day 1 to day 12, and SHH was supplied from day 4 to day 12. 2i administration decreased *EN1* and *CORIN* while increasing *FOXG1* and *SOX1* expression (Figures 6F and 6G). More importantly, *LHX6* and *LHX8* were now expressed at a later stage (Figure 6H), suggesting that an MGE fate was induced. Again, the same results were obtained from both H1 hESCs and hiPSCs (Figures S5H–S5L).

### Specification of Different Neuronal Subtypes with AD and EB Paradigms

**Figure 7:**
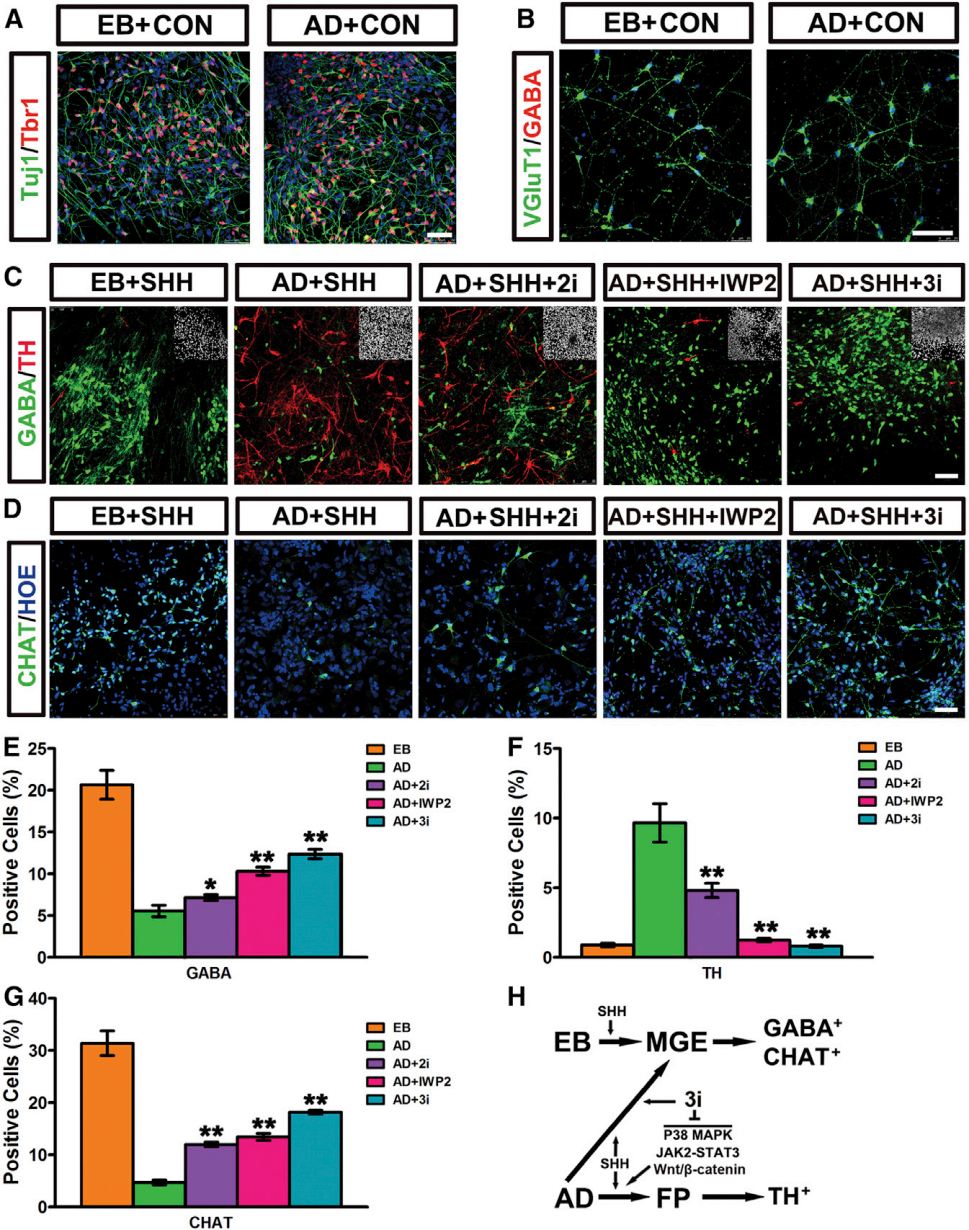
Generation of Distinct Neuronal Subtypes with AD and EB Paradigms (A and B) Without SHH treatment, both EB and AD anterior-dorsal progenitors generate TUJ1^+^/TBR1^+^ (A) and VGluT1^+^/GABA^−^ (B) cortical neurons at day 50 post differentiation. Scale bars, 50 μm. (C and D) EB-MGE progenitors mostly generate GABA interneurons (C) and CHAT^+^ cholinergic neurons (D). AD-FP progenitors mostly generate TH^+^ DA neurons (C). Blocking p38, STAT3, and Wnts pathways generates more GABA and cholinergic but fewer DA neurons. Insets show Hoechst counterstaining of nuclei. Scale bars, 50 μm. (E–G) Quantification of percentage of positive GABA (E), TH (F), and CHAT (G) cells in (C) and (D). Data are presented as mean ± SEM of three independent experiments. Unpaired two-tailed Student's t test: ^∗^p < 0.05, ^∗∗^p < 0.01. (H) Summary of strategies for differentiation of various ventral progenitors and related neuronal subtypes via EB and AD differentiation paradigms in combination with small molecules. See also Figure S6.

During development, MGE progenitors yield GABA interneurons and basal forebrain cholinergic neurons, while the FP progenitors almost exclusively generate DA neurons. For terminal neuronal differentiation, we digested neuroprogenitors and plated them on laminin precoated coverslips. After continued differentiation for 4 weeks, TBR1^+^/VGluT1^+^ cortical excitatory neurons were generated in both EB and AD differentiation paradigms without SHH treatment (Figures 7A and 7B). In vitro generated EB-MGE progenitors ventralized via SHH treatment differentiated into GABA neurons and CHAT^+^ cholinergic neurons after continued differentiation (Figures 7C–7G). In contrast, upon SHH patterning, the AD-FP progenitors preferentially generated DA neurons (Figures 7C and 7F). 2i treatment in the AD cells resulted in the generation of more GABA and CHAT^+^ neurons and fewer DA neurons, coinciding with an FP to MGE fate switch (Figures 7C–7G). *β-catenin* KO or applying IWP2 to block Wnts signaling behaved similarly to 2i treatment (Figures 7C–7G and S6). Notably, blocking all three signaling pathways simultaneously (*β-catenin* KO plus 2i, or 3i) generated a maximum amount of GABA and CHAT^+^ neurons of MGE origin, which reached an efficiency of about 60% compared with the EB-MGE cells (Figures 7C–7H and S6).

## Discussion

In this study we present evidence and show that hPSCs mostly adopt a rostral-dorsal identity during in vitro neural differentiation with both EB and AD paradigms in the absence of patterning morphogens. SHH patterns in vitro differentiated human neuroectoderm under both EB and AD conditions to ventral progenitors, as demonstrated by a *PAX6* to *NKX2.1* expression shift. However, differential ventral fates are acquired in hPSCs under EB versus AD conditions in response to SHH stimulation (Figure 7H). An MGE regional fate followed by GABA and cholinergic neurons will be generated in SHH-treated EB cells, while ventralized AD cells mostly yield FP progenitors and TH^+^ neurons. Wnts/β-catenin, STAT3, and p38 pathways are crucial for proper FP specification, and blocking these pathways could largely switch FP to MGE (Figure 7H).

Fetal mesencephalic or striatal tissues have been used as a source of DA neurons or GABA neurons for transplantation in clinical trials (Lindvall et al., 1989, Philpott et al., 1997). These clinical studies raise the hope of curing hitherto intractable human neurodegenerative diseases through replacement therapy. Recent advances in targeted differentiation of hPSCs to neuronal subtypes have begun to confirm their therapeutic potential in epilepsy, Huntington disease, Parkinson's disease, and Alzheimer's disease (Cunningham et al., 2014, Liu et al., 2013, Ma et al., 2012, Roy et al., 2006, Yue et al., 2015). Obtaining functional neuronal subtypes is the major roadblock in regenerative medicine for treating these neurological disorders. Here, we provide a framework for the generation of distinct ventral neuronal subtypes of either FP or MGE origin through a combination of differentiation paradigms and small molecules. These human MGE and FP progenitors as well as their differentiated progeny will thus serve as invaluable cellular sources for the study of brain development and related diseases.

## Experimental Procedures

### HPSC Culture

hESC lines H1 and H9 (WA 01 and WA09, passages 25–45, WiCell Agreement No. 14-W0377 and 17-W0044) and hiPSCs were cultured on a feeder layer of irradiated MEFs as previously described (Chi et al., 2016, Liu et al., 2016a, Thomson et al., 1998, Zhang et al., 2010). Cells were expanded every 5 days through dispase (Gibco, 17105) digestion. The components of the hESC culture medium (hESCM) are DMEM/F12, 20% knockout serum replacer, 1× minimum essential medium non-essential amino acids solution, 1× L-glutamine solution, 0.1 mM β-mercaptoethanol, and 4 ng/mL fibroblast growth factor 2.

### EB Formation and Neural Differentiation

The detailed procedures of the EB differentiation system were described previously (Zhang et al., 2001, Zhang and Zhang, 2010). For caudalization, RA (1 μM, Sigma) was added to the neuroectoderm cells from day 10 to day 17. For ventralization, a combination of SHH (250 ng/mL, R&D Systems) and the smoothened activator purmorphamine (0.3 μM, Stemgent), a condition referred to hereafter as SHH, was added to the neuroectoderm cells from day 10 to day 17.

### Dual-Smad Inhibition-Based AD Culture and Neural Differentiation

The AD differentiation system was modified from the previously published protocols (Chambers et al., 2009, Xi et al., 2012). hPSCs at approximately 10% confluence (1 day after passaging) were cultured in hESCM/NIM (50%:50%) supplied with SB431542 (2 μM, Stemgent) and LDN193189 (200 nM, Stemgent) for the first 7 days. RA or SHH were added to the cells at an indicated time point after differentiation, or on day 4 after differentiation if not mentioned specifically.

### Statistical Analysis

Data were analyzed using Student's t test for comparison of independent means with pooled estimates of common variances.

## Author Contributions

C.L., F.B., G.L., L.L., and Z.X. conceived the study. C.L. and F.B. performed most of the experiments. L.Z., F.Y., C.Z., R.X., X.X., and M.L. set up the homologous recombination system in hPSCs. Z.K., D.Y., L.S., and J.C. performed the bioinformatics analyses. C.L. and F.B. collected and analyzed the data. C.L., F.B., L.L., and Z.X. wrote the manuscript.

## Supplemental Information

Document S1. Supplemental Experimental Procedures and Figures S1–S6

Document S2. Article plus Supplemental Information
