# Targeted Differentiation of Regional Ventral Neuroprogenitors and Related Neuronal Subtypes from Human Pluripotent Stem Cells

**DOI:** 10.1016/j.stemcr.2026.103055

**Published:** 2026-07-30

**Authors:** Liankai Chi, Beibei Fan, Kunshan Zhang, Yanhua Du, Zhongliang Liu, Yujiang Fang, Zhenyu Chen, Xudong Ren, Xiangjie Xu, Cizhong Jiang, Siguang Li, Lin Ma, Liang Gao, Ling Liu, Xiaoqing Zhang

## Main text

(Stem Cell Reports *7*, 941–954; November 8, 2016)

In the original version of this article, there were unintended errors in Figure 1A and Figure 6A. The leftmost image in Figure 1A is related to a PAX6/SOX1 expression pattern in neural progenitors differentiated from human pluripotent stem cells in suspension culture. However, the image we used is from the day 14 differentiation derivatives, but not day 17, although PAX6 and SOX1 exhibit the same expression patterns at these two time points. The first image in Figure 6A is related to FOXA2 expression in the control group. Although the pattern is correct (no FOXA2 expression at this condition), the image itself is not the one from our original experimental record. The revised Figure 1 and Figure 6 with correct panels now appear below. We, the authors, sincerely apologize for any confusion that these inadvertent errors may have caused. No other correction to the text or figure legend was needed. These errors do not alter the results or conclusions of the study.

**Figure:**
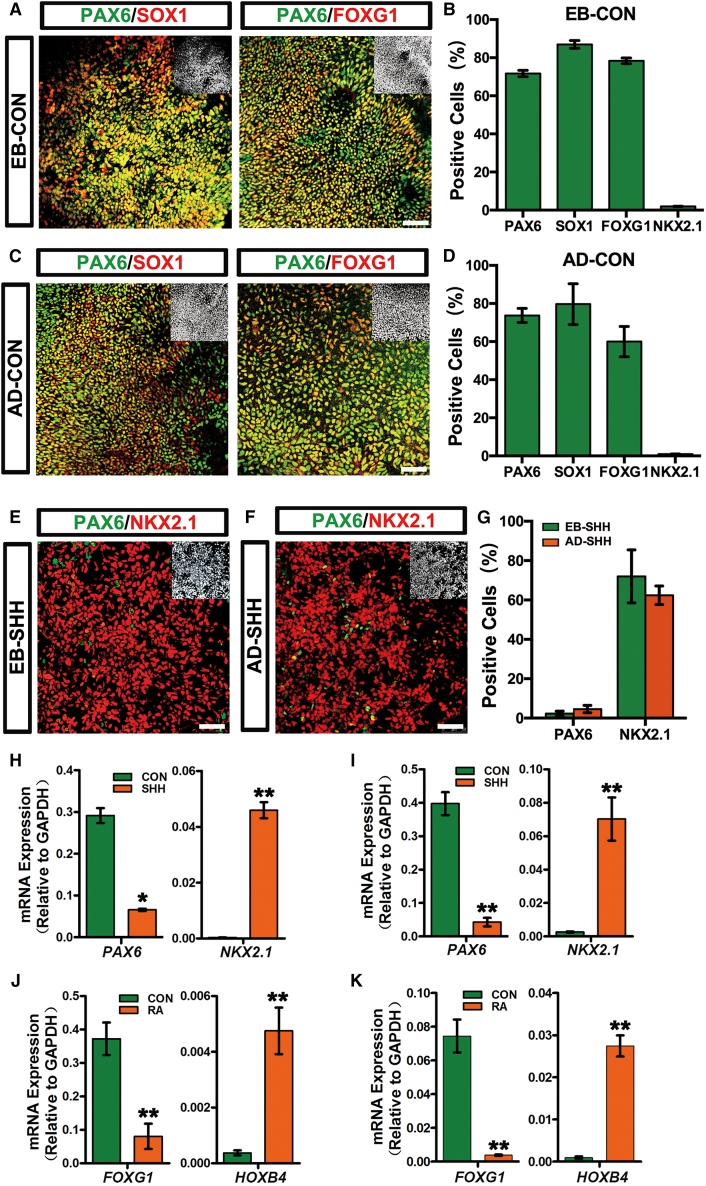
Figure 1. Neuroectoderm Generated with Both EB and AD Paradigms Holds Normal Patterning Potency along the A-P and D-V Axes

**Figure:**
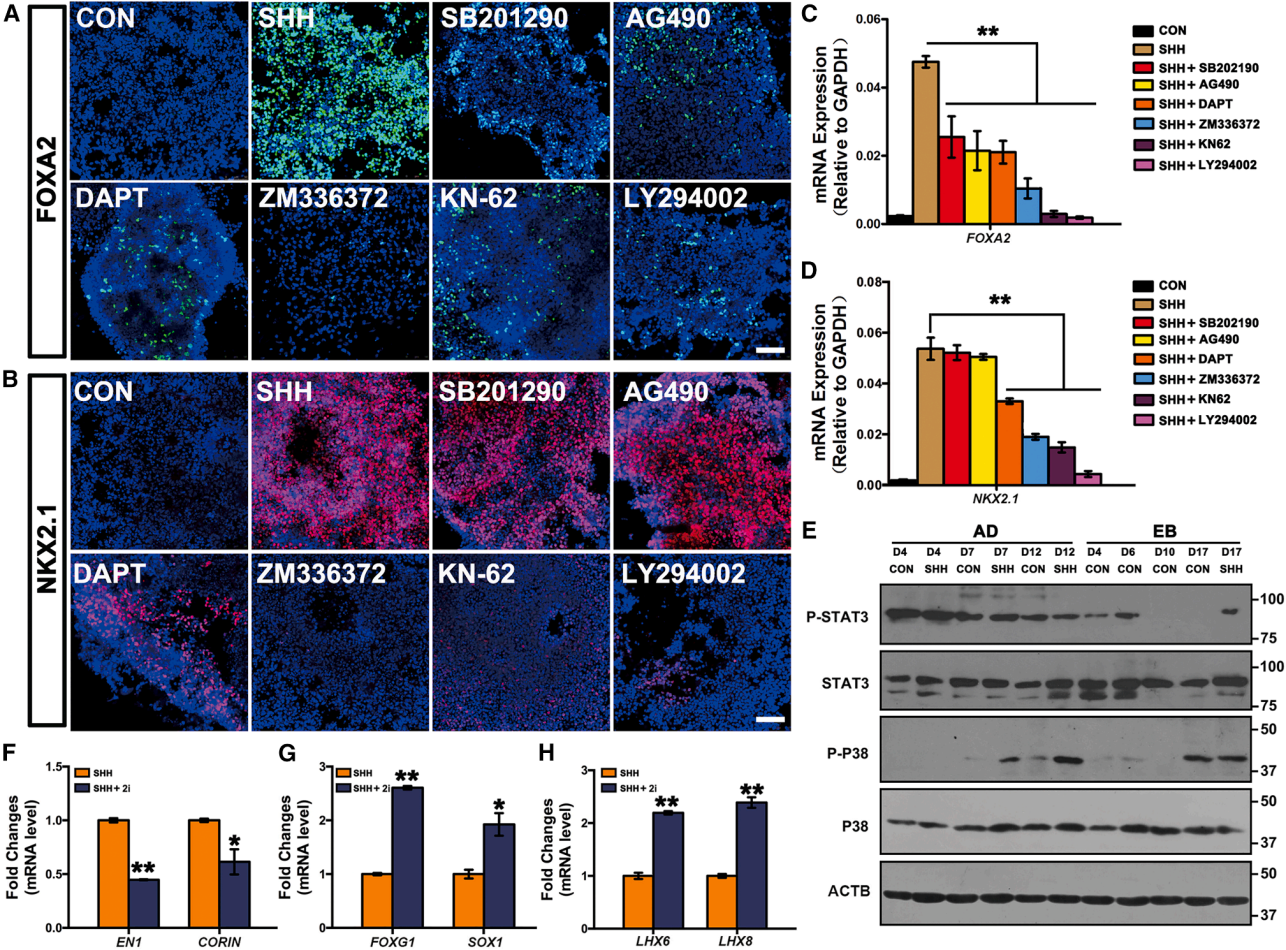
Figure 6. The STAT3 and p38 MAPK Signaling Pathways Are Crucial for Floor-Plate Specification

