# Supplementary material for: Targeted Differentiation of Regional Ventral Neuroprogenitors and Related Neuronal Subtypes from Human Pluripotent Stem Cells
Source: Stem Cell Reports. 2016 Oct 6;7(5):941–54. doi: 10.1016/j.stemcr.2016.09.003 (PMC5106484; doi:10.1016/j.stemcr.2016.09.003)
Supplement: Document S1. Supplemental Experimental Procedures and Figures S1–S6 [file mmc1.pdf]

Stem Cell Reports, Volume 7

Supplemental Information

Targeted Differentiation of Regional Ventral Neuroprogenitors and Related Neuronal Subtypes from Human Pluripotent Stem Cells

Liankai Chi, Beibei Fan, Kunshan Zhang, Yanhua Du, Zhongliang Liu, Yujiang Fang, Zhenyu Chen, Xudong Ren, Xiangjie Xu, Cizhong Jiang, Siguang Li, Lin Ma, Liang Gao, Ling Liu, and Xiaoqing Zhang

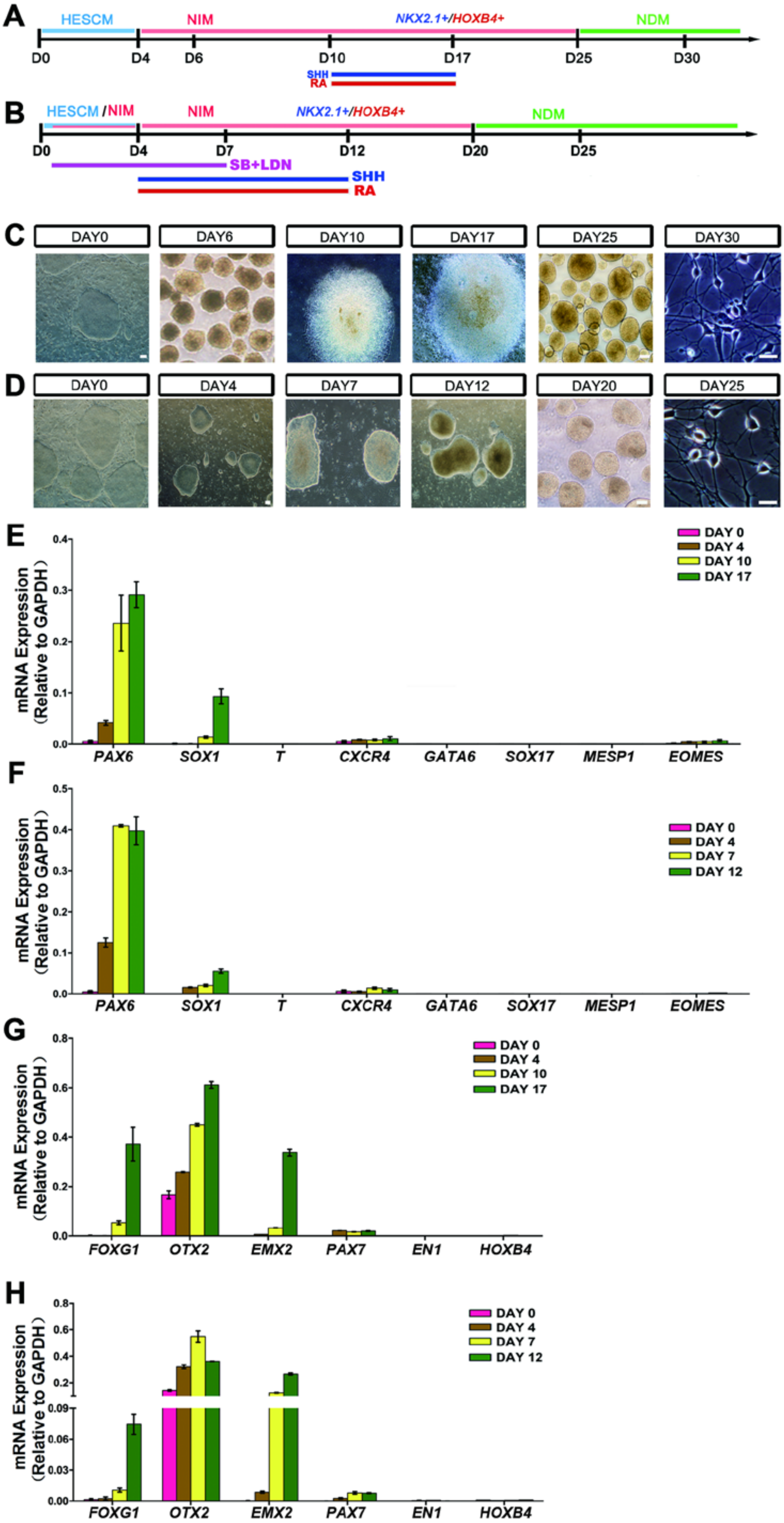
A
HESCM
NIM
NKX2.1+/HOXB4+
NDM
D0
D4
D6
D10
D17
D25
D30
SHH
RA
B
HESCM /NIM
NIM
NKX2.1+/HOXB4+
NDM
D0
D4
D7
D12
D20
D25
SB+LDN
SHH
RA
C
DAY0
DAY6
DAY10
DAY17
DAY25
DAY30
D
DAY0
DAY4
DAY7
DAY12
DAY20
DAY25
E
mRNA Expression (Relative to GAPDH)
DAY 0
DAY 4
DAY 10
DAY 17
PAX6
SOX1
T
CXCR4
GATA6
SOX17
MESP1
EOMES
F
mRNA Expression (Relative to GAPDH)
DAY 0
DAY 4
DAY 7
DAY 12
PAX6
SOX1
T
CXCR4
GATA6
SOX17
MESP1
EOMES
G
mRNA Expression (Relative to GAPDH)
DAY 0
DAY 4
DAY 10
DAY 17
FOXG1
OTX2
EMX2
PAX7
EN1
HOXB4
H
mRNA Expression (Relative to GAPDH)
DAY 0
DAY 4
DAY 7
DAY 12
FOXG1
OTX2
EMX2
PAX7
EN1
HOXB4

Figure S1. Both EB and AD neural differentiation paradigms are highly efficient and favor of generating anterior neural progenitors in the absence of exogenous morphogens, related to Figure 1.

(**A and B**) Overview of the protocol for the EB (A) and the AD (B) differentiation paradigms. HESCM, human ESCs culture medium; NIM, neural induction medium; NDM, neural differentiation medium.

(**C and D**) Bright field images of differentiation derivatives at different time points under EB (C) and AD (D) conditions. For EB cells in (C), Day 0, embryonic stem cells stage; Day 6-10, neuroectoderm; Day 17-25, regional progenitors; Day 30, neurons. For AD cells in (D), Day 0, embryonic stem cells stage; Day 4-7, neuroectoderm; Day 12-20, regional progenitors; Day 25, neurons. Scale bars, 50 μm.

(**E and F**) The mRNA expression of neuroectoderm, mesoderm and endoderm genes during differentiation with either EB (E) or AD (F) differentiation paradigms without patterning morphogens. Data are presented as mean±SEM of three independent experiments.

(**G and H**) The mRNA expression of forebrain, midbrain and hindbrain genes during differentiation with either EB (G) or AD (H) differentiation paradigms without patterning morphogens. Data are presented as mean±SEM of three independent experiments.

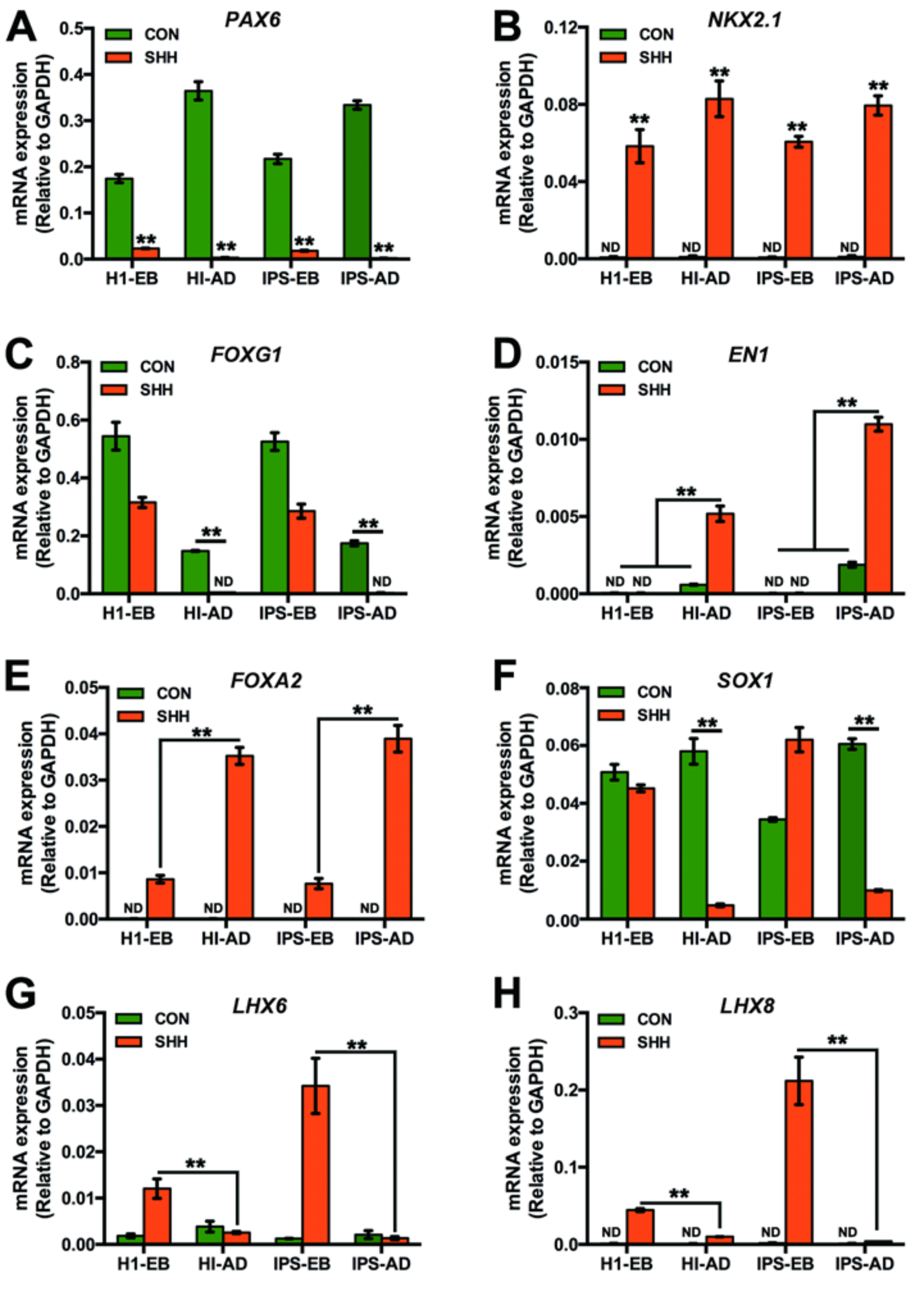
A
PAX6
B
NKX2.1
C
FOXG1
D
EN1
E
FOXA2
F
SOX1
G
LHX6
H
LHX8
CON
SHH
mRNA expression (Relative to GAPDH)
H1-EB
HI-AD
IPS-EB
IPS-AD
ND
**

Figure S2. H1 hESCs and hiPSCs are conserved with H9 hESCs in yielding similar regional neuroprogenitors under AD and EB conditions, related to Figure 2.

(**A-H**) *PAX*6, *NKX2.1*, *FOXG*1, *EN*1, *FOXA2*, *SOX*1, *LHX*6 and *LHX*8 mRNA expression in day 17 cells differentiated with the EB protocol or day 12 cells differentiated with the AD protocol. Under the control conditions, no SHH is supplied. Under the SHH treated conditions, SHH is added from day 10-17 in EB or day 4-12 in AD cells. Data are presented as mean±SEM of three independent experiments. Unpaired two-tailed Student's t-test. ** $p<0.01$.

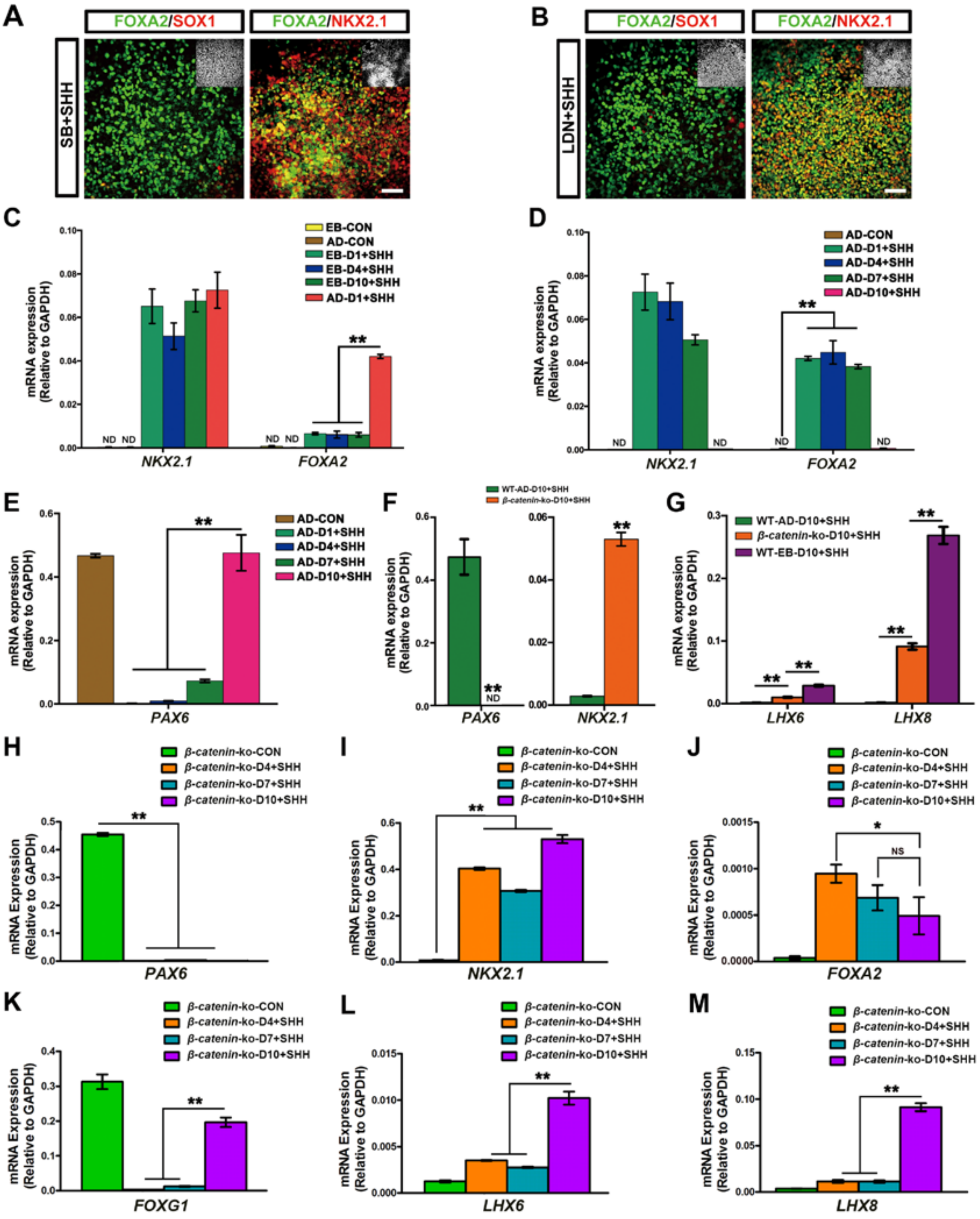
A
B
FOXA2/SOX1
FOXA2/NKX2.1
SB+SHH
LDN+SHH
C
D
E
F
G
H
I
J
K
L
M
mRNA expression (Relative to GAPDH)
mRNA Expression (Relative to GAPDH)
EB-CON
AD-CON
EB-D1+SHH
EB-D4+SHH
EB-D10+SHH
AD-D1+SHH
AD-D4+SHH
AD-D7+SHH
AD-D10+SHH
WT-AD-D10+SHH
β-catenin-ko-D10+SHH
WT-EB-D10+SHH
β-catenin-ko-CON
β-catenin-ko-D4+SHH
β-catenin-ko-D7+SHH
β-catenin-ko-D10+SHH
ND
NS
NKX2.1
FOXA2
PAX6
LHX6
LHX8
FOXG1

Figure S3. Effects of dual Smads inhibition, timing of SHH exposure or Wnts/β-catenin signaling on FP specification with the AD differentiation paradigm, related to Figure 3.

(**A and B**) Removal of LDN193189 or SB431542 in the AD differentiation protocol does not affect FP specification. Scale bars, 50 μm.

(**C**) Advanced SHH exposure (day1, day4 vs day 10) with EB differentiation paradigm could not induce a FP fate as shown by low *FOXA2* while high *NKX*2.1 mRNA expression. Data are presented as mean±SEM of three independent experiments. Unpaired two-tailed Student's t-test. ** $p<0.01$.

(**D**) FP fate is persistently induced under the AD differentiation conditions even when SHH is treated at different time points, including day1, day4 and day7. Cells at day10 are fixed to the dorsal identity since neither *NKX*2.1 nor *FOXA2* could be induced by SHH. Data are presented as mean±SEM of three independent experiments. Unpaired two-tailed Student's t-test. ** $p<0.01$.

(**E**) AD cells at day10 are committed to the dorsal identity since *PAX*6 could no longer be repressed by SHH. Data are presented as mean±SEM of three independent experiments. Unpaired two-tailed Student's t-test. ** $p<0.01$.

(**F**) In *β-catenin* KO hESCs, SHH treatment at day10 represses *Pax6* while induces *NKX2.1* mRNA expression under the AD culture conditions. Data are presented as mean±SEM of three independent experiments. Unpaired two-tailed Student's t-test. ** $p<0.01$.

(**G**) In *β-catenin* KO hESCs, SHH treatment at day10 AD cells moderately induces *LHX6* and *LHX8* mRNA expression. However, the levels of *LHX6* and *LHX8* are still significantly lower as compared with EB-MGE cells. Data are presented as mean±SEM of three independent experiments. Unpaired two-tailed Student's t-test. ** $p<0.01$.

(**H-I**) SHH treatment at day 4, 7 or 10 efficiently ventralizes the *β-catenin* KO AD cells as shown by *PAX6* repression and *NKX2.1* induction. Data are presented as mean±SEM of three independent experiments. Unpaired two-tailed Student's t-test. ** $p<0.01$.

(**J-M**) SHH treatment at day10, but not at day 4 or day 7, partially converts FP to MGE in *β-catenin* KO AD cells as evidenced by low *FOXA2*, while high *FOXG1*, *LHX6* and *LHX8* expression. Data are presented as mean±SEM of three independent experiments. Unpaired two-tailed Student's t-test. * $p<0.05$; ** $p<0.01$.

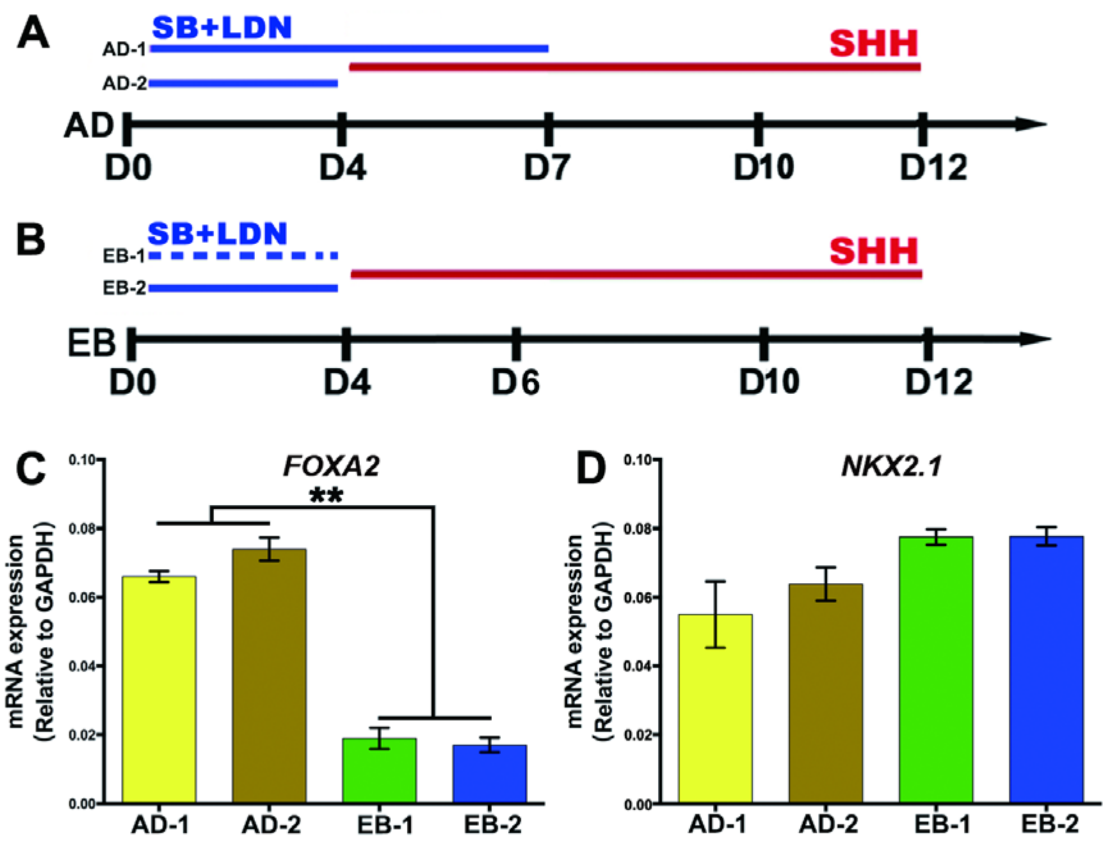

Figure S4. Modifying AD and EB differentiation paradigms for transcriptome profiling, related to Figure 4.

(**A**) Under the AD conditions, SB431542 and LDN193189 are supplied to the cells from day1-4 or day 1-7, while SHH is added at day 4 and thereafter.

(**B**) Under the EB conditions, cells are treated with or without SB431542 and LDN193189 for 4 days followed by SHH addition from day 4 to day 12 for ventralization.

(**C and D**) The mRNA levels of *FOXA2* and *NKX2.1* in day 12 differentiated cells from either AD or EB groups. Data are presented as mean±SEM of three independent experiments. Unpaired two-tailed Student's t-test. ** $p<0.01$.

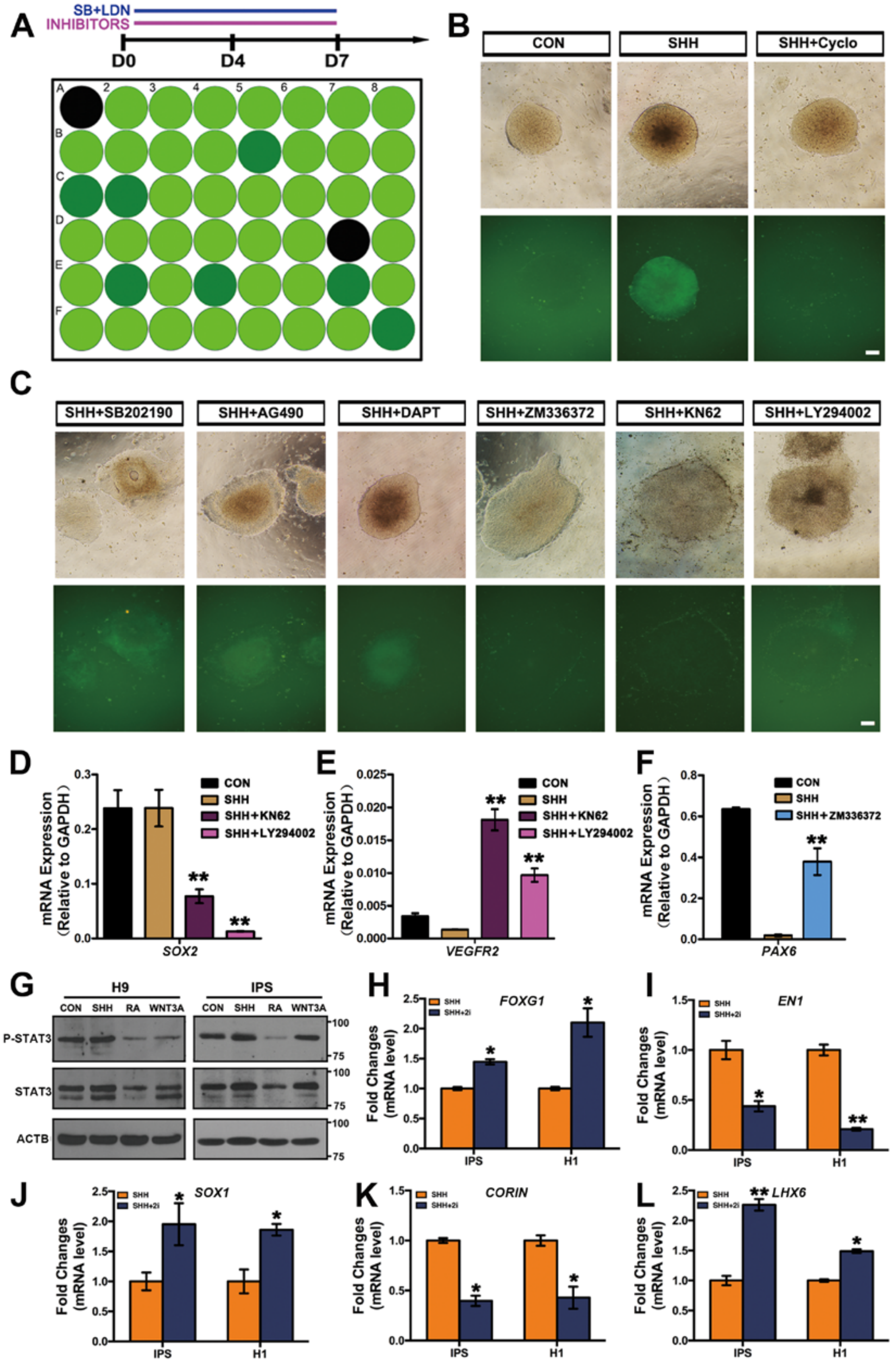
A
SB+LDN
INHIBITORS
D0
D4
D7
B
CON
SHH
SHH+Cyclo
C
SHH+SB202190
SHH+AG490
SHH+DAPT
SHH+ZM336372
SHH+KN62
SHH+LY294002
D
mRNA Expression (Relative to GAPDH)
CON
SHH
SHH+KN62
SHH+LY294002
SOX2
E
VEGFR2
F
SHH+ZM336372
PAX6
G
H9
IPS
CON SHH RA WNT3A
P-STAT3
STAT3
ACTB
H
FOXG1
Fold Changes (mRNA level)
SHH
SHH+2i
IPS
H1
I
EN1
J
SOX1
K
CORIN
L
LHX6

Figure S5. Small molecule screening identifies the beneficial roles of the STAT3 and the p38 MAPK signaling inhibitors in specifying MGE, related to Figure 6.

(**A**) Screening protocol used for the present study. SHH, SB431542, LDN193189 and candidate small molecules are added on day 1 post AD differentiation and GFP expression is analyzed on day 7.

(**B and C**) Representative hits that repress GFP expression induced by SHH. Cyclopamine is one of the hits and also serves as a positive control. Scale bars, 50 μm.

(**D and E**) Decreased expression of *SOX2* (D) and increased expression of *VEGFR2* (E) after KN62 or LY294002 treatment. Data are presented as mean ±SEM of three independent experiments. Unpaired two-tailed Student's t-test. ** $p<0.01$.

(**F**) ZM336372 treatment abrogates downregulation of *PAX6* induced by SHH. Data are presented as mean ±SEM of three independent experiments. Unpaired two-tailed Student's t-test. ** $p<0.01$.

(**G**) Western blot shows RA treatment decreases total STAT3 and phospho-STAT3 protein levels in H9 hESCs and hiPSCs under AD conditions at day 4.

(**H-L**) Q-PCR results show that combined AG490 and SB202190 treatment decreases *EN1*(I), *CORIN* (K), but increases *FOXG1*(H), *SOX1* (J) and *LHX6* (L) expression in SHH patterned H1 hESCs and hiPSCs under the AD conditions. Data are presented as mean±SEM of three independent experiments. Unpaired two-tailed Student's t-test. * $p<0.05$; ** $p<0.01$.

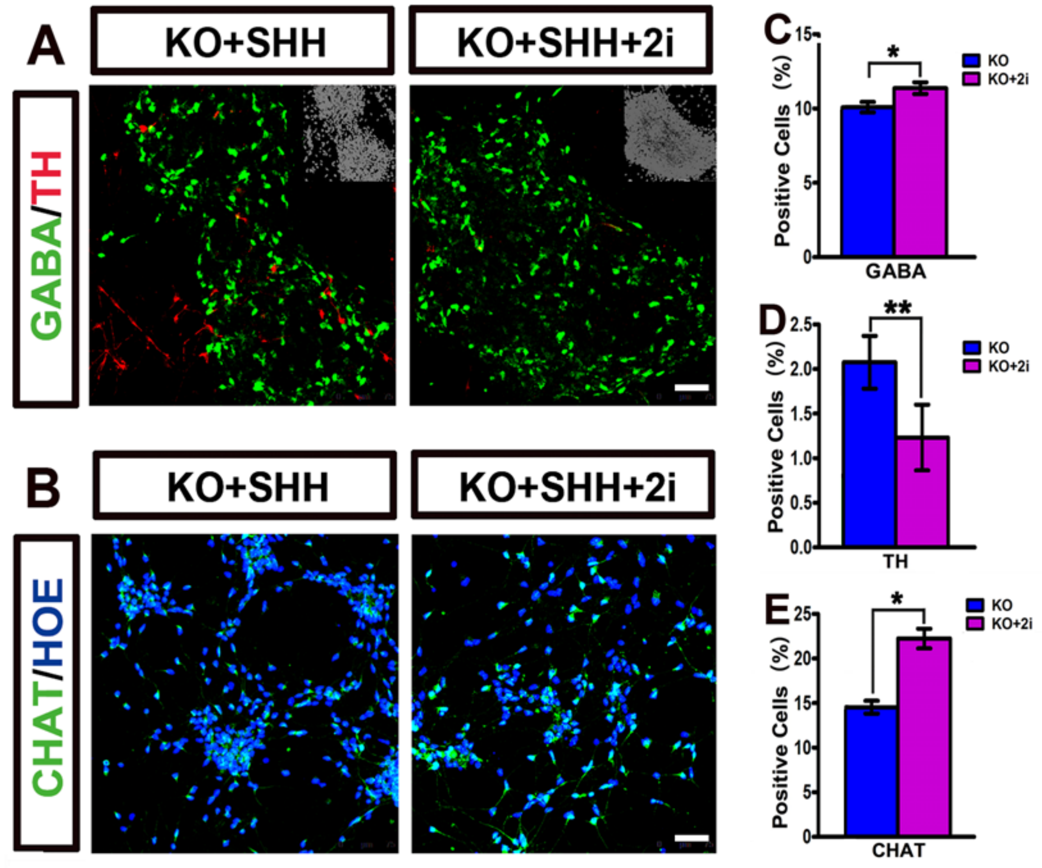

Figure S6. STAT3 and p38 MAPK signaling inhibition facilitates MGE-related GABA and cholinergic neuron generation under AD conditions, related to Figure 7.

(**A and B**) Confocal images show more GABA+ and CHAT+, while less TH+ neurons are generated in β-catenin KO AD cells in the presence of AG490 and SB202190. Insets show hoechst counterstaining of nuclei. Scale bars, 50 μm.

(**C-E**) Quantification of percentage of positive cells in A and B. Data are presented as mean±SEM of three independent experiments. Unpaired two-tailed Student's t-test. * $p<0.05$; ** $p<0.01$.

Supplementary Methods

mRNA extraction and qRT-PCR

Total RNA was isolated by using the Trizol kit (Invitrogen) and RNA concentration was determined by NanoDrop 2000c (Thermo Scientific). 1μg of total RNA from each sample was reverse transcribed into cDNA using SuperScript III (Invitrogen) and subjected to quantitative RT-PCR (qRT-PCR, Bio-Rad, CFX Connect Real-Time System) using the Ssofast EvaGreen kit (Bio-Rad). The housekeeping gene glyceraldehyde-3-phosphate dehydrogenase (GAPDH) was amplified as an internal control in gene expression analysis. Primer oligonucleotides used for qRT-PCR were as follows:

GENE	Forward Primer	Reverse Primer
GAPDH	ATGACATCAAGAAGGTGGTG	CATACCAGGAAATGAGCTTG
PAX6	TCTTTGCTTGGGAAATCCG	CTGCCCGTTCAACATCCTTAG
NKX2.1	AACCAAGCGCATCCAATCTCAAG	TGTGCCCAGAGTGAAGTTTGGTC
FOXG1	AGAAGAACGGCAAGTACGAGA	TGTTGAGGGACAGATTGTGGC
EN1	GGACAATGACGTTGAAACGCAGC	AAGGTCGTAAGCGGTTTGGCTAG
HOXB4	AAAGAGCCCGTCGTCTACC	GTGTAGGCGGTCCGAGAG
SOX1	GCGGAAAGCGTTTTCTTTG	TAATCTGACTTCTCCTCCCT
FOXA2	GGAGCAGCTACTATGCAGAGC	CGTGTTCATGCCGTTCATCC
LHX6	GGGCGCGTCATAAAAAGCAC	TGAACGGGGTGTAGTGGATGT
LHX8	GGACAACAACCCAGATGC	GTGGAGGATGAGTGATTAGGA
T	ACAGCCAGCAACCTGGGTA	CATGCAGGTGAGTTGTCAGAA
SOX17	ATACGCCAGTGACGACCAG	GCGGCCGGTACTTGTAGTT
VEGFR2	TAGAAGGTGCCCAGGAAAAG	CAAGTAGCCTGTCTTCAGTTC
ZCCHC12	CTGGGGAGAAGTCTCGGTC	GGCCCCTAAGGGTTTTCATCA
COUP-TFII	GTGCCAACAGGAACTGTCCC	AGTGCGTACTGGCCTGGATT
SOX2	GCCCTGCAGTACAACTCCAT	TGGAGTGGGAGGAAGAGGTA
FOXA1	GCAATACTCGCCTTACGGCT	TACACACCTTGGTAGTACGCC
CXCR4	CCACGCCACCAACAGTCAGA	GGCAAAGATGAAGTCGGGAAT
GATA6	GTGAACTGCGGCTCCATC	GTGTGACAGTTGGCACAGGA
MESP1	CTCTGTTGGAGACCTGGATG	CCTGCTTGCCTCAAAGTG
EOMES	AAATGGGTGACCTGTGGCAAAG	CTCCTGTCTCATCCAGTGGGAA
OTX2	GACCCGGTACCCAGACATC	TGGCCACTTGTTCCACTCTC
EMX2	CGGCACTCAGCTACGCTAAC	CAAGTCCGGGTTGGAGTAGAC
PAX7	GACGACGGCGAAAAGAAGG	GTAGTGGGTCCTCTCAAAGGC
WNT5A	ATTCTTGGTGGTCGCTAGGTA	CGCCTTCTCCGATGTACTGC
RAX	GGCTGTACACGTCCGGGTAG	CCGGCGAAGCGAAACTGTCA
CHL1	ACCAACATTTTCGTGGACTAAG	TCTGACATAGCGATTCCCAGTT
CTIP2	CTCCGAGCTCAGGAAAGTGTC	TCATCTTTACCTGCAATGTTCT
FOXP2	AATGTGGGAGCCATACGAAG	GCCTGCCTTATGAGAGTTGC

CORIN	CCTCCTCCGGTTCCTATTGC	CCAAAGGTTCACTCCCATTTGA
SPC	CACCTGAAACGCCTTCTTATCG	TTTCTGGCTCATGTGGAGACC
TG	AGACACCTCCTACCTCCCTCA	TCCTTGGACATCGCTTTGGC

Immunocytochemistry and imaging

Immunofluorescent staining on coverslip cultures was described previously (Yang et al., 2008). Coverslip cultures were fixed immediately in 4% paraformaldehyde for 10 min at room temperature. After adequate washing with PBS, cells were incubated in a blocking buffer (10% donkey serum plus 0.2% Triton X-100 in PBS) for 60 min at room temperature followed by overnight primary antibody incubation at 4°C. On the next day, coverslips were washed with PBS and stained with the fluorescently conjugated secondary antibodies (1:1000, Jackson, West Grove, PA). Nuclei were counterstained with Hoechst 33258. Coverslips were visualized with Leica TSC SP5 (Leica Microsystems, Bensheim, Germany) confocal laser-scanning microscope. Antibodies used in this study included PAX6 (1:1,000, mouse IgG, Developmental Studies Hybridoma Bank, AB528427), PAX6 (1:1,000, rabbit IgG, Covance, PRB-278P), NKX2.1 (1:400, mouse IgG, Chemicon, MAB5460), SOX1 (1:1,000, goat IgG, R&D, AF3369), SOX2 (1:1,000, goat IgG, R&D, AF2018), FOXA2 (1:1,000, rabbit IgG, Abcam, ab108422), FOXG1 (1:500, rabbit IgG, Abcam, ab23470), β-III tubulin (1:1,000, mouse IgG, Sigma, T8660), GABA (1:1,000, rabbit IgG, Sigma, A2052), TH (1:5,000, mouse IgG, Sigma, T2928), VGluT1(1:200, mouse IgG, Millipore, ab5905), TBR1(1:1,000, mouse IgG, Abcam, ab31940), CHAT (1:400, goat IgG, Chemicon, MAB5270).

Western blotting

Cells were collected at different time points and lysed in RIPA buffer. Protein concentrations were calculated with the BCA kit (Thermo Scientific). After denaturing, 30μg of total proteins were subjected to SDS-PAGE. Antibodies used are FOXA2 (1:5,000, rabbit IgG, Abcam, ab108422), P38 MAPK (1:1000, rabbit IgG, Cell Signaling, 9212)，Phospho-p38 MAPK (1:1000, rabbit IgG, Cell Signaling, 9211), STAT3 (1:2000, mouse IgG, Cell Signaling, 9132), Phospho-STAT3 (1:1000, rabbit IgG, Cell Signaling, 9131), ACTB (1:5000, mouse IgG, Sigma, A5316).

RNA-Seq and data analysis

Total RNA was prepared from EB (EB, EB with SB431542 and LDN193189) and AD (AD with SB431542 and LDN193189) cells at day 4 with Trizol kit (Invitrogen). Three biological replicates per group were used. Trimmed reads were aligned to the reference hg19 using TopHat 2 and Bowtie 0.12.8. Transcript abundances were quantified using Cufflinks 2.2.0. Expression levels were estimated for known RefSeq genes using counts of reads across genic features. Weighted gene correlation network analysis (WGCNA) was performed as described before (Langfelder and Horvath, 2008).

Fisher exact test was used to score GO (Gene Ontology) terms for significant enrichment. The raw data were deposited in the NCBI's Sequence Read Archive (accession number, GSE82052).

CRISPR/Cas9-mediated *FOXA2*$^{eGFP/W}$ hESCs reporter line construction

The *FOXA2* targeting donor vector comprised an 858bp 5'homology arm, eGFP preceded by a P2A sequence, loxP flanked PGK promoter driven puromycin resistance gene and an 846bp 3' homology arm (Fig.5A). The detailed information of the Cas9 expressing vector and gRNA could be found in the paper published by Mali et al (Mali et al., 2013). The *FOXA2* gRNA targeting sequence was AAGCCGTCGTCTTCTTAAG. 1μg Cas9 plasmid and 1μg gRNA were transiently cotransfected into HEK293FT cells (Invitrogen) by using the calcium phosphate precipitation method to assess the targeting efficiency of gRNA. hESCs were pretreated with 1 mM Y27632, a Rho Kinase (ROCK)-inhibitor (Calbiochem), for 4 hours, digested into single cells with trypsin and electroporated with 40 μg *FOXA2* donor plasmid, 5 μg of Cas9 and 5 μg gRNA targeting plasmids. Cells were then plated on MEF feeder layer supplied with Y27632 and cultured overnight. Medium was changed on the next day and individual colonies were selected with puromycin (1 mg/ml) for 10–14 days. Positive colonies were identified by genomic DNA PCR using the following primers:

Forward-1(F1): ATCAACAACCTCATGTCCTCGGAGC;

Reverse-1(R1): TTCCCGTTTTCCTCCTTATATAGA；

Reverse-2(R2): ACACGCTGAACTTGTGGCCGTTTAC.

FOXA2 knockout cell lines construction

The *FOXA2* targeting donor vector comprised an 864bp 5' homology arm, followed by Ef1α promoter driven BSD resistance gene and an 850bp 3' homology arm. The gRNA targeting sequence was AGCAACATGAACGCCGGCC.

Human ESCs were pretreated with 1 mM Y27632 for at least 3 hours, digested into single cells with trypsin and electroporated with 5 μg of Cas9, 5 μg gRNA targeting plasmids and 40 μg targeting donor plasmid. Cells were then plated on MEF feeder layer supplied with Y27632 and cultured overnight. Medium was changed on the next day and individual colonies were selected with blasticidin (1 mg/ml). Positive colonies were identified by genomic DNA PCR.

For amplifying the 5' arm and 3' arm of *FOXA2*, the following primer sets were used:

FOXA2-5'arm-BsmBI-F:

AACGTA cgtctcagatc TCGCTCCTTCCCCAACCTCAGGACC;

FOXA2-5'arm-BsmBI-R:

CTTGAC cgtctccctagc CAGTCTCCGGACTCCGAGTCTGTTT;

FOXA2-3'arm-BsaI-F:

GTCATA ggtctcagaca CAAGGGAGAAGAAATCCATAACACC;

FOXA2-3'arm-BsaI-R:

GCTGAC ggtctcaggcc TGAAGATAGATGGACAAGTATGTGA.

Primer sets for identitying the wild type and recombinated alleles were as follows:

FOXA2-Forward-wt: ATCAACAACCTCATGTCCTCGGAGC;

FOXA2-Reverse-wt: TTCCCGTTTTCCTCCTTATATAGAA;

FOXA2-Forward-hr: GGAGCAGCTACTATGCAGAGC;

FOXA2-Reverse-hr: CTCGGGGACTGTGGGCGATGTGCGC.

Lentivirus production and transduction of *EEF1A1*-rtTA hESCs

For inducible overexpression, *FOXG1* cDNA was PCR amplified and subsequently constructed into the pLVX-Tight-Puro vector (Clontech, USA) through BamHI and EcoRI sites. For lentivirus production, 10 μg lentiviral transfer vector, 7.5 μg Δ8.9 and 5 μg VSVG plasmids were cotransfected into HEK 293FT cells (Invitrogen) cultured in a 10 cm dish using the calcium phosphate precipitation method. Sixteen hours post transfection, fresh cell culture medium were supplied. After 2 days, the medium containing viral particles was collected and filtered through a 0.45μm filter (Millipore). The viral particles were further concentrated by ultracentrifugation (SW28 rotor, Beckman) at 50,000 g for 2.5 hours. The pellet was resuspended in hESCM. For transduction, *EEF1A1*-rtTA cell line (Chi et al., 2016) were passaged normally and pelleted by brief centrifugation. Cell pellets were then incubated with 100 μl of concentrated virus (10^6 transducing units/ml) at 37°C for 30 minutes. The virus and cell mixture were then transferred to a MEF feeder layer overnight and fresh medium was supplied on the next day.

Primer sets for amplifying *FOXG1* were as follows:

FOXG1-BamHI-F:

ACGT ggatcc GACGACTGGGTGATGCTGGAACATGGGAGATAG GAAA;

FOXG1-EcoRI-R:

ACGT gaattc TTAATGTATTAAAGGGTTGGAAGAA.

Supplemental References

Chi, L., Fan, B., Feng, D., Chen, Z., Liu, Z., Hui, Y., Xu, X., Ma, L., Fang, Y., Zhang, Q., *et al.* (2016). The Dorsoventral Patterning of Human Forebrain Follows an Activation/Transformation Model. Cereb Cortex *doi: 10.1093/cercor/bhw152*.

Langfelder, P., and Horvath, S. (2008). WGCNA: an R package for weighted correlation network analysis. BMC bioinformatics *9*, 559.

Mali, P., Yang, L., Esvelt, K.M., Aach, J., Guell, M., DiCarlo, J.E., Norville, J.E., and Church, G.M. (2013). RNA-guided human genome engineering via Cas9. Science *339*, 823-826.

Yang, D.L., Zhang, Z.J., Oldenburg, M., Ayala, M., and Zhang, S.C. (2008). Human embryonic stem cell-derived dopaminergic neurons reverse functional deficit in Parkinsonian rats. Stem cells *26*, 55-63.
